# Physical Adsorption of Graphene Oxide onto Polymer Latexes and Characterization of the Resulting Nanocomposite Particles

**DOI:** 10.1021/acs.langmuir.2c00327

**Published:** 2022-06-30

**Authors:** Shang-Pin Wen, Elisabeth Trinh, Qi Yue, Lee A. Fielding

**Affiliations:** † Department of Materials, School of Natural Sciences, University of Manchester, Oxford Road, Manchester M13 9PL, U.K.; ‡ Henry Royce Institute, The University of Manchester, Oxford Road, Manchester M13 9PL, U.K.

## Abstract

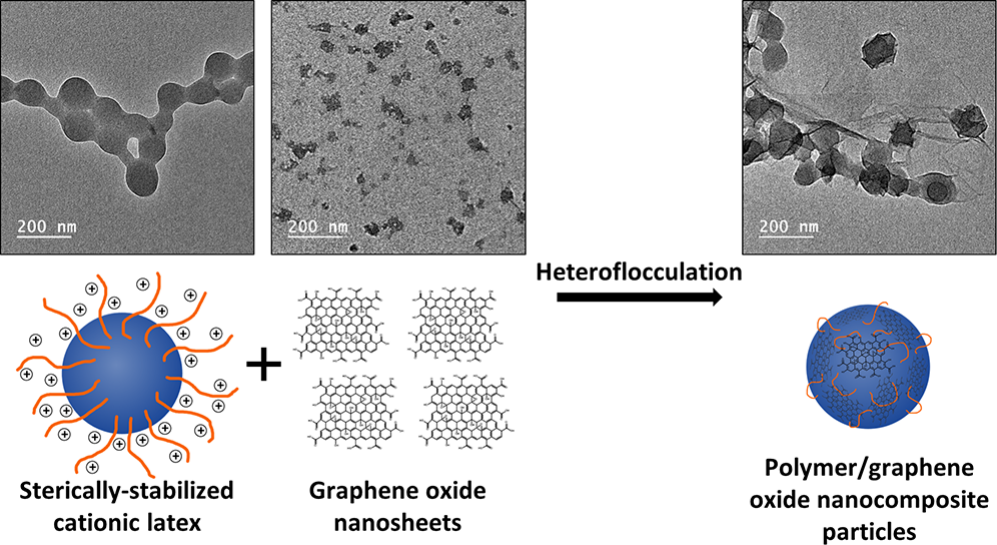

Polymer/graphene oxide (GO) nanocomposite particles were prepared *via* heteroflocculation between 140–220 nm cationic latex nanoparticles and anionic GO nanosheets in either acidic or basic conditions. It is demonstrated that nanocomposite particles can be formed using either poly(2-vinylpyridine)-*b*-poly(benzyl methacrylate) (P2VP–PBzMA) block copolymer nanoparticles prepared by reversible-addition chain-transfer (RAFT)-mediated polymerization-induced self-assembly (PISA), or poly(ethylene glycol)methacrylate (PEGMA)-stabilized P2VP latexes prepared by traditional emulsion polymerization. These two latexes are different morphologically as the P2VP–PBzMA block copolymer latexes have P2VP steric stabilizer chains in their corona, whereas the PEGMA-stabilized P2VP particles have a P2VP core and a nonionic steric stabilizer. Nevertheless, both the P2VP–PBzMA and PEGMA-stabilized P2VP latexes are cationic at low pH. Thus, the addition of GO to these latexes causes flocculation to occur immediately due to the opposite charges between the anionic GO nanosheets and cationic latexes. Control heteroflocculation experiments were conducted using anionic sterically stabilized poly(potassium 3-sulfopropyl methacrylate)-*b*-poly(benzyl methacrylate) (PKSPMA–PBzMA) and nonionic poly(benzyl methacrylate) (PBzMA) nanoparticles to demonstrate that polymer/GO nanocomposite particles were not formed. The degree of flocculation and the strength of electrostatic interaction between the cationic polymer latexes and GO were assessed using disc centrifuge photosedimentometry (DCP), transmission electron microscopy (TEM), and UV–visible spectrophotometry. These studies suggest that the optimal conditions for the formation of polymer/GO nanocomposite particles were GO contents between 10% and 20% w/w relative to latex, with the latexes containing P2VP in their corona having a stronger electrostatic attraction to the GO sheets.

## Introduction

Nanocomposite particles have attracted extensive attention from both academic and industrial researchers in the past two decades.^1−5^ In particular, nanocomposite particles comprising polymer and graphene have received much attention.^6,7^ Graphene is a two-dimensional material with exceptional thermal, mechanical, and electrical properties.^8^ These exceptional properties afford tremendous possibilities for the design of advanced materials with potential applications, such as sensors,^9^ electrode materials,^10^ catalytic materials,^11^ and supercapacitors.^12^ However, graphene has a relatively hydrophobic surface with high van der Waals attraction between graphene sheets. This leads to the tendency for irreversible stacking-induced aggregation, hindering production, processing, and storage for either research or industrial manufacturing.^13^

Graphene oxide (GO) has attracted attention as it is a material chemically derived from graphene.^14^ GO is commonly prepared *via* modified Hummers–Offeman methods by oxidation of graphite using strong and concentrated oxidizing acids (e.g., H_2_SO_4_ and HNO_3_).^15,16^ This process results in oxygen-containing functional groups (e.g., carboxylic, hydroxyl, and epoxy groups) being created and covalently attached to the basal carbon plane. Specifically, carboxylic functionalities are mostly located at the sheet edges, whereas hydroxyl and epoxide functional groups are on the top and bottom surfaces of the GO sheets.^17^ The presence of these functional groups significantly disturbs the planar graphene structure and facilitates exfoliation to generate single-layer GO sheets as a dispersion in aqueous media.^18^ Due to ionization of the carboxylic acid and phenolic hydroxyl groups attached to the carbon skeleton, GO sheets are negatively charged across a wide pH range, with the ζ potential becoming more negative as the pH increases.^19,20^ Furthermore, these oxygen-containing groups on the GO surface can be functionalized for the preparation of polymer/GO nanocomposites.

Numerous approaches have been reported for the preparation of graphene-based polymer nanocomposites using graphene-related materials (e.g., graphene, graphene oxide, and reduced graphene oxide) as a filler *via* solution blending^21^ or melt processing.^22^ GO is usually functionalized through the carboxylic or hydroxyl groups on the basal surface *via* esterification,^23^ amination,^24^ isocyanate grafting,^25^ or polymer grafting.^26^ For solution blending, the polymer is dissolved in a selected solvent, and the GO nanofillers are dispersed in the polymer solution. Generally, more homogeneous nanocomposites can be obtained, but the residual solvents in nanocomposites are hard to remove.^21^ In contrast, from an industrial standpoint, melt processing is potentially preferred, as it is direct and can be conducted without using solvents, and thus is suitable for a wide range of polymers and nanofillers.^27^ However, this approach needs to be conducted at a relatively high temperature (>180 °C)^28^ and the nanofillers readily aggregate due to high surface areas, leading to poor dispersion and phase separation of the polymer/inorganic phase.^29^

Recently, it has been shown that GO-based polymer nanocomposites can be readily prepared through hybrid latex particles on the nanoscale *via* heteroflocculation between negatively charged GO and positively charged polymer latex nanoparticles.^30−32^ Pham et al. prepared poly(methyl methacrylate)/GO (PMMA/GO) nanocomposites by heteroflocculation between positively charged PMMA latex (∼200 nm) and negatively charged GO sheets.^30^ Wu et al.^33^ reported polystyrene/GO (PS/GO) nanocomposites obtained by heteroflocculation between amine-modified PS latex (150 to 220 nm) and negatively charged GO sheets. In those studies, the GO sheets were much larger than the size of the PMMA or PS nanoparticles, leading to several latex nanoparticles being wrapped by one large GO sheet. Both the PMMA/GO and PS/GO nanocomposites were further dried and hot-pressed to obtain composite pellets. These pellets exhibited excellent electrical conductivity. In contrast, Hong et al. demonstrated PS/rGO nanocomposite particles with core/shell morphology prepared *via* a layer-by-layer heteroflocculation route using negatively charged PS latexes (∼1.2 μm), positively charged rGO^–^NH_3_^+^, and negatively charged rGO^–^COO^–^ sheets.^34^ The thickness of the rGO shell could be increased by alternating coatings of rGO-NH_3_^+^/rGO-COO^–^ layers.

However, the studies above were mainly focused on the surface morphologies of the nanocomposite particles and their bulk electric, thermal, or mechanical properties. It is noteworthy that those reported polymer/GO nanocomposites were prepared using either GO sheets or latex particles at nanosize. Furthermore, to the best of our knowledge, there are no prior reports on investigating the details of the electrostatically induced heteroflocculation process between GO nanosheets and polymer latex nanoparticles prepared *via* RAFT-mediated PISA.

**Scheme 1:**
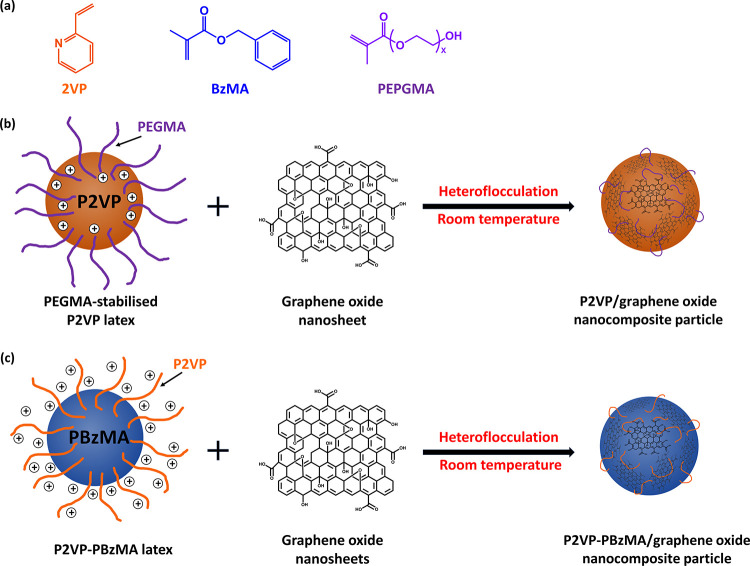
(a) Chemical Structures of 2-Vinylpyridine (2VP), Benzyl Methacrylate (BzMA), and Poly(ethylene glycol) Methacrylate (PEGMA), and Schematic Representation of the Physical Adsorption of Graphene Oxide Nanosheets onto Sterically Stabilized (b) PEGMA-Stabilized P2VP and (c) P2VP–PBzMA Latexes *via* Electrostatically Induced Heteroflocculation

Herein, the preparation of polymer/GO nanocomposite particles *via* electrostatically induced heteroflocculation is reported (Scheme 1). Specifically, a cationic poly(ethylene glycol) methacrylate (PEGMA)-stabilized P2VP latex^35,36^ and P2VP-stabilized poly(benzyl methacrylate) (PBzMA) latexes^37^ were synthesized *via* conventional and RAFT emulsion polymerization, respectively. Polymer/GO nanocomposite particles were prepared *via* heteroflocculation at room temperature by mixing these positively charged latex nanoparticles and the negatively charged GO nanosheets. It is noteworthy that both the latexes and GO sheets used herein were at the nanoscale. Furthermore, anionic sterically stabilized poly(potassium 3-sulfopropyl methacrylate)-poly(benzyl methacrylate) (PKSPMA–PBzMA)^38^ and nonionic poly(benzyl methacrylate)^39^ latexes were used to perform control heteroflocculation experiments. The polymer latexes and resulting polymer/GO nanocomposite particles were characterized *via* dynamic light scattering (DLS), disc centrifuge photosedimentometry (DCP), transmission electron microscopy (TEM), UV–visible spectrophotometry (UV–vis), and aqueous electrophoresis. Furthermore, both the PEGMA-stabilized P2VP and P2VP–PBzMA latexes are pH-responsive and have different surface charges at varying pH. Thus, the effects of solution pH on the formation of the polymer/GO nanocomposite particles prepared *via* heteroflocculation were investigated, and it is shown that heteroflocculation can be achieved in either acidic (pH 2) or basic (pH 9) conditions.

## Experimental Section

### Materials

2-Vinylpyridine (2VP, 97%) and divinylbenzene (DVB, 80 mol % 1,4-divinyl content) were purchased from Sigma-Aldrich (U.K.) and Fluka (U.K.), respectively, and passed through a column of activated basic alumina to remove inhibitors and impurities before use. 2,2′-azodiisobutyramidine dihydrochloride (AIBA, 97%) and monomethoxy-capped poly(ethylene glycol) methacrylate (PEGMA) macromonomer (*M*_n_ = 2000 g mol^–1^, *M*_w_/*M*_n_ = 1.10; 50% w/w in H_2_O) were purchased from Sigma-Aldrich (U.K.) and used as received. Aliquat 336 (99.9%) and dialysis tubing (regenerated cellulose, molecular weight cutoff (MWCO) = 12 kDa, diameter = 16 mm) were purchased from Fisher Scientific (U.K.) and used as received. Graphene oxide aqueous dispersion (monolayer content >95%; concentration 4 mg mL^–1^) was purchased from Graphenea (Spain) and purified by dialysis against water to remove impurities before use. Deionized water was used in all experiments.

Anionic poly(potassium 3-sulfopropyl methacrylate)-poly(benzyl methacrylate) (PKSPMA–PBzMA), nonionic poly(benzyl methacrylate) (PBzMA), cationic poly(2-vinylpyridine)-poly(benzyl methacrylate) (P2VP–PBzMA), and cationic PEGMA-stabilized P2VP latexes were prepared in-house according to previously reported protocols.^35−39^ For the sake of brevity, a shorthand label is used throughout this manuscript: PEGMA-stabilized P2VP and P2VP_x_-PBzMA_y_ latexes are denoted as “PEGVP” and “V_x_-B_y_,” respectively.

### Preparation of Polymer/GO Nanocomposite Particles *via* Heteroflocculation

Aqueous dispersions of GO and latex particles were diluted separately using deionized water. The solutions were adjusted to pH 2, 5, or 9 using HCl or KOH and then water was added to adjust the solids content to 0.1% w/w. An appropriate volume of the latex particle dispersion was added to the GO dispersion with stirring using IKA vortex mixer for 60 s. In a typical example, 2500 μL of 0.1% w/w V_32_-B_300_ latex dispersion was added to 500 μL of 0.1% w/w GO dispersion to form 3 mL of a 0.1% w/w nanocomposite particle dispersion. Samples were allowed to equilibrate using a roller mixer at room temperature for 48 h.

### Characterization

#### Dynamic Light Scattering

DLS studies were performed using a Malvern Zetasizer Nano ZS instrument equipped with a He–Ne solid-state laser operating at 633 nm and back-scattered light at a scattering angle of 173°. Latex dispersions were diluted to approximately 0.1% w/w using deionized water. Samples were analyzed at 25 °C using disposable plastic cuvettes, and data were averaged over three consecutive measurements.

#### Aqueous Electrophoresis

Aqueous electrophoresis studies were performed using the same Malvern Zetasizer Nano ZS instrument described above. For analysis of the PEGVP latexes and GO, the solution was initially adjusted to pH 11 using KOH in the presence of 1.0 mM KCl and then manually decreased to pH 2 by addition of HCl as required. For analysis of the V_x_-B_y_ latexes, the solution was initially adjusted to pH 2 using HCl in the presence of 1.0 mM KCl and then manually increased to pH 11 by addition of KOH as required. Aqueous dispersions (approximately 0.1% w/w) were analyzed at 25 °C using disposable folded capillary cells (Malvern DTS1017), and data were averaged over three consecutive measurements.

#### Gravimetry

Monomer conversions were determined by gravimetry. Aliquots were withdrawn and weighed (approximately 1.0 g) in 7 mL vials. After weighing, the samples were immediately quenched with approximately 10 μL of 1% w/w hydroquinone in an ice-water bath. The specimens were placed in an oven and dried at 60 °C to constant weight. Conversions were calculated from the measured dry residue.

#### Transmission Electron Microscopy

TEM images were recorded using a FEI Tecnai G2 20 instrument operating at an accelerating voltage of 200 kV and connected to a Gatan 1k CCD camera. Samples for TEM observation were prepared by depositing 2 μL of diluted samples (approximately 0.1% w/w) onto 400 mesh carbon-coated copper grids. For PEGVP latexes and polymer/GO nanocomposite particles, the samples were dried overnight at ambient temperature. For TEM studies of the V_x_-B_y_ nanoparticles, the grids were dried for 30 min at ambient temperature and then carefully blotted with filter paper to remove excess solution. The samples were stained in a vapor space above ruthenium tetroxide (RuO_4_) solution for 7 min at ambient temperature.^40^ The mean nanoparticle diameters were determined using ImageJ software, and over 200 randomly selected particles were measured for each sample.

#### UV–Visible Spectrophotometry

UV spectra were recorded using an Agilent Cary 60 UV–vis spectrophotometer between 200 and 800 nm with a scan speed of 600 nm min^–1^. Samples were prepared by centrifuging the heteroflocculation dispersions at 200 rpm for 5 min, and subsequently, the supernatants were carefully collected. A moderate centrifuge speed was utilized to ensure that only polymer/GO nanocomposite particles settled to the bottom and free GO remained dispersed in the supernatant. The supernatants were diluted to approximately 0.05% w/w using deionized water. UV–vis samples were analyzed at room temperature using quartz cuvettes. The concentration of free GO in the supernatant was calculated using the Beer–Lambert law. The calibration samples for GO were prepared at concentrations ranging from 1.0 × 10^–3^ to 6.7 × 10^–2^ mg mL^–1^ and analyzed at room temperature.

#### Disc Centrifuge Photosedimentometry

Particle size distributions were determined *via* DCP studies using a CPS DC24000 instrument operating at 22,000 rpm. The spin fluid was built using 12.0 to 4.0% w/w aqueous sucrose, and *n*-dodecane (0.5 mL) was injected to avoid surface evaporation and extend the lifetime of the gradient. The aqueous sucrose solutions were adjusted to pH 2, 5, or 9 using HCl or KOH before use to match the pH of the dispersion being studied. The disc centrifuge was calibrated using a 348 nm polystyrene latex standard.

## Results and Discussion

### Characterization of P2VP–PBzMA and PEGMA-Stabilized P2VP Latexes

**Figure 1:**
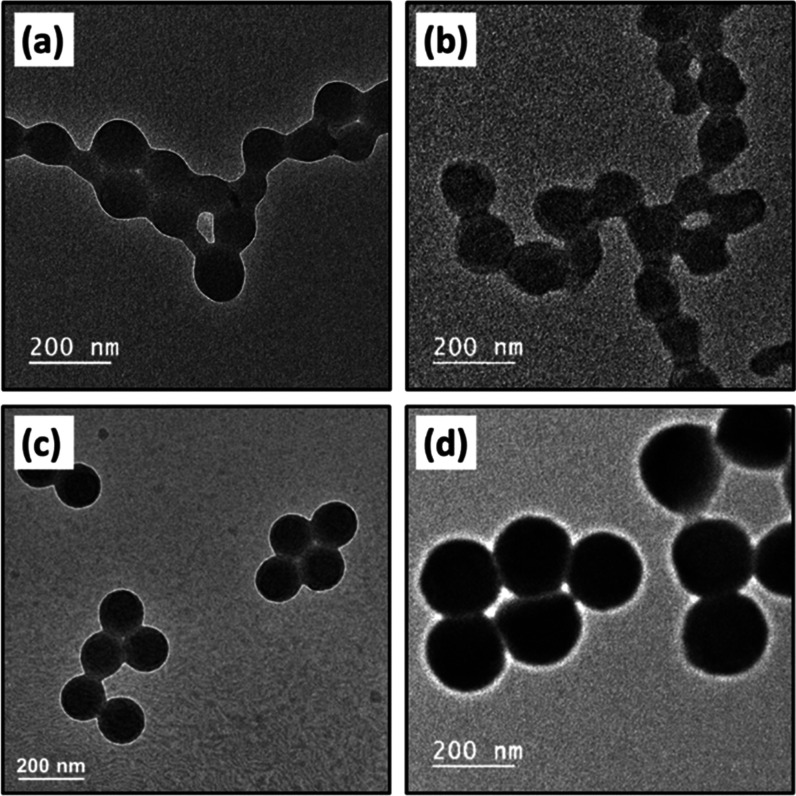
Representative TEM images for (a) P2VP_32_–PBzMA_300_, (b) P2VP_67_–PBzMA_300_, (c) PKSPMA_32_–PBzMA_300_, and (d) PEGMA-stabilized P2VP latex nanoparticles. Panels (a–d) represent entries 1–4 in Table 1, respectively.

Two P2VP_x_–PBzMA_300_ (V*_x_*-B_300_) latexes with different P2VP stabilizer chain lengths and a PEGMA-stabilized P2VP (PEGVP) latex were prepared by RAFT emulsion and conventional emulsion polymerization, respectively (Table 1). The preparation of V_x_-B_y_ latexes *via* RAFT-mediated PISA using P2VP as a macro-CTA has recently been discussed in detail by our group.^37^ Briefly, V_x_-B_y_ diblock copolymer nanoparticles with controllable particle diameters can be obtained *via* RAFT emulsion polymerization using the same target copolymer composition at varying solution pH. Herein, two V_x_-B_300_ latexes with the same core-forming PBzMA block target degree of polymerization (DP) and different P2VP chain lengths were prepared. Specifically, P2VP_32_–PBzMA_300_ (V_32_–B_300_) and P2VP_67_–PBzMA_300_ (V_67_–B_300_) diblock copolymer nanoparticles were prepared *via* RAFT emulsion polymerization of BzMA at pH 2.5 and 3.0, respectively. In both cases, high monomer conversions (>99%) were achieved after polymerization at 70 °C for 24 h, as determined *via* gravimetry. Figure S1a,b shows that both the V_32_-B_300_ and V_67_-B_300_ latexes had relatively narrow particle size distributions with hydrodynamic diameters of 139 and 149 nm, respectively. TEM images were consistent with DLS analysis and confirmed that these latexes were near-monodisperse (Figure 1a,b).

**Table 1:** Summary of the Sterically Stabilized Latexes Used in This Work

entry^a^	target composition	conversion^b^ (%)	*D*_h_^c^ (nm)	*D*_TEM_^d^ (nm)
1	P2VP_32_–PBzMA_300_	99	139 (0.098)	88 ± 7
2	P2VP_67_–PBzMA_300_	99	149 (0.057)	102 ± 8
3	PKSPMA_32_–PBzMA_300_	99	177 (0.040)	132 ± 4
4	PEGMA-stabilized P2VP	96	222 (0.052)	173 ± 5
5	PBzMA	96	289 (0.051)	234 ± 21

a Entries 1 and 2 were prepared *via* RAFT aqueous emulsion polymerization at 70 °C using P2VP as a macromolecular chain-transfer agent (macro-CTA) and at pH 2.5 and 3.0, respectively. Entry 3 was prepared *via* RAFT emulsion polymerization at 70 °C using PKSPMA as a macro-CTA in a methanol/water mixture at an alcohol content of 33% w/w. Entry 4 was prepared *via* conventional emulsion polymerization at 60 °C using 10% w/w nonionic PEGMA stabilizer, 10% w/w Aliquot 336 surfactant, and 1% w/w DVB cross-linker. Entry 5 was synthesized *via* RAFT miniemulsion polymerization at 70 °C at a dispersed phase concentration of 20% w/w and using 2.4% w/w hexadecane and 7.8% w/w Lutensol TO 20 relative to BzMA ([BzMA]:[PETTCCP]:[AIBN] = 600:2:1).

b Monomer conversions were determined *via* gravimetry.

c Mean hydrodynamic diameter obtained *via* DLS, where DLS polydispersity index values are indicated in brackets.

d Mean TEM particle diameters were calculated by analyzing 200 particles using ImageJ software.

PEGMA-stabilized P2VP (PEGVP) latexes with controllable diameters can be prepared *via* conventional aqueous emulsion polymerization by altering the monomer and initiator concentration.^35,36,41^ According to a previously reported protocol,^36^ a PEGVP latex with a target hydrodynamic diameter of approximately 200 nm was prepared by conducting the polymerization at total solids content of 11.0% with 0.2% w/w AIBA initiator and 1.0% w/w DVB as cross-linker relative to monomer, respectively. After polymerization for 24 h, high monomer conversion (96%) was achieved, and all excess stabilizer (PEGMA) and surfactant (Aliquat 336) were removed by dialysis against water and three centrifugation/redispersion cycles. Figure S1d shows that the PEGVP latex had a relatively narrow particle size distribution, with a hydrodynamic diameter of 222 nm, as confirmed by TEM studies (Figure 1d).

Figure S2a shows the ζ potential as a function of pH for the PEGVP latex (entry 4, Table 1). The PEGVP particles were slightly negatively charged at pH 11, with a ζ potential of approximately −3 mV. As the solution pH was lowered by the addition of HCl, the ζ potential became more positive and reached a plateau value of approximately +25 mV at pH 4.1. This is in good agreement with reported p*K*_a_ values, ranging from 3.85 to 4.75, for P2VP latexes with different degrees of cross-linking.^35^ A similar trend was observed for the V_32_–B_300_ (Figure S3a) and V_67_–B_300_ latexes (Figure S4a). The slightly negative ζ potentials of PEGVP and V_32_–B_300_ latexes at high pH can be attributed to the adsorption of OH^–^ ions on the primarily uncharged surface.^42,43^ It is noteworthy that the ζ potentials of the V_32_–B_300_ and V_67_–B_300_ latexes were much higher than that of the PEGVP latex across the whole pH range studied. This is because the P2VP chains are present in the corona of the V_x_-B_300_ latexes, whereas they are in the core of the PEGVP latexes and are surrounded by nonionic PEGMA stabilizer.

Figure S2b shows the mean hydrodynamic diameter (*D*_h_) as a function of pH for the PEGVP latex (Entry 4, Table 1). No obvious particle diameter change was observed between pH 11 and 5. However, the particle diameter increased significantly below pH 4.1. This can be attributed to the pyridine groups on P2VP chains becoming protonated and inducing swelling of the lightly cross-linked latex particles to form microgels. Interestingly, the observed particle diameter trend was the opposite for V_32_–B_300_ latexes (Figure S3b) and V_67_–B_300_ latexes (Figure S4b), with the particle diameters increasing significantly above pH 5. As the P2VP chains do not form the core of the particle, no latex-to-microgel transition occurs. These observations can be attributed to the relatively high ionic strength (K^+^ and Cl^–^) generated from the solution pH (HCl and KOH) and background electrolyte (KCl) inducing particle flocculation.^37^ Briefly, at high pH, the degree of protonation of the P2VP stabilizer decreases, resulting in a weaker positive charge and lower electrostatic repulsion among particles. Furthermore, the relatively high ionic strength screens any residual electrostatic repulsion and induces flocculation,^44,45^ resulting in the large particle diameters reported by DLS analysis. It is noteworthy that the induced flocculation can be avoided by diluting these latexes using water at the desired pH directly to minimize the buildup of ionic strength.^37^ Therefore, in this work, all of the latex dispersions utilized for heteroflocculation with GO nanosheets were directly diluted using water at the corresponding pH with no added electrolyte.

### Characterization of the Commercial Graphene Oxide Dispersion

A commercial graphene oxide (GO) aqueous dispersion was used in this work. Generally, GO is prepared *via* oxidation of graphite flakes using strong concentrated acid (e.g., HNO_3_ and H_2_SO_4_). This process results in hydroxyl (−OH) and epoxy groups being formed on the basal planes and carboxyl (−COOH) groups present on the sheet edges of the graphite structure to form GO (Scheme S1).^14^ Furthermore, the NO^3–^ and SO_4_^2–^ are inserted into the graphene layers, and the interlayer spacing of the graphite structure is exfoliated to form GO sheets.^46^ However, the GO sheets may still tend to congregate and form multilayer agglomerates during storage.^47^

Normally, oxidized graphite is readily exfoliated using ultrasonication to generate GO nanosheets.^48,49^ To obtain relatively uniform GO nanosheets, the commercial GO aqueous dispersion (4 mg mL^–1^) was sonicated using an ultrasonic probe at various amplitudes (70 or 90%) and process times (5, 10, or 30 min, see Table S1). Although GO sheets are nonspherical, the mean hydrodynamic diameter determined *via* DLS analysis can still be used for qualitative quantification of the changes in GO size.^50,51^ DLS reported that the diameter of GO after sonication decreased significantly from approximately 1500 to 230–430 nm (Figure S5 and Table S1). Furthermore, the reported diameter of GO decreased with increasing process time at a fixed amplitude. For example, the diameter was approximately 395 nm after ultrasonication at 70% amplitude for 5 min, whereas the diameter was 235 nm (about 41% less) for 30 min. However, the GO diameters did not become increasingly smaller when using higher amplitude (90%) for a fixed process time. For instance, after ultrasonication for 10 min, the diameter was 340 nm when using 70% amplitude, whereas the diameter was 375 nm when using 90% amplitude. Furthermore, the degree of aggregation of GO after ultrasonication was monitored *via* DLS (Figure S5). In all cases, only minor increases in particle diameter were observed during storage for 3 days.

Aqueous electrophoresis measurements performed on the commercial GO aqueous dispersion after sonication at 70% aptitude for 30 min (entry 4, Table S1) as a function of pH are shown in Figure S6. The measured ζ potential of GO between pH 2 and 12 was −18 to −38 mV, which is in agreement with the reported values in the literature.^18,52^ As pH increases, the GO becomes more negative due to the deprotonation of carboxylic acid on the sheet edges (Scheme S1).^53^

**Figure 2:**
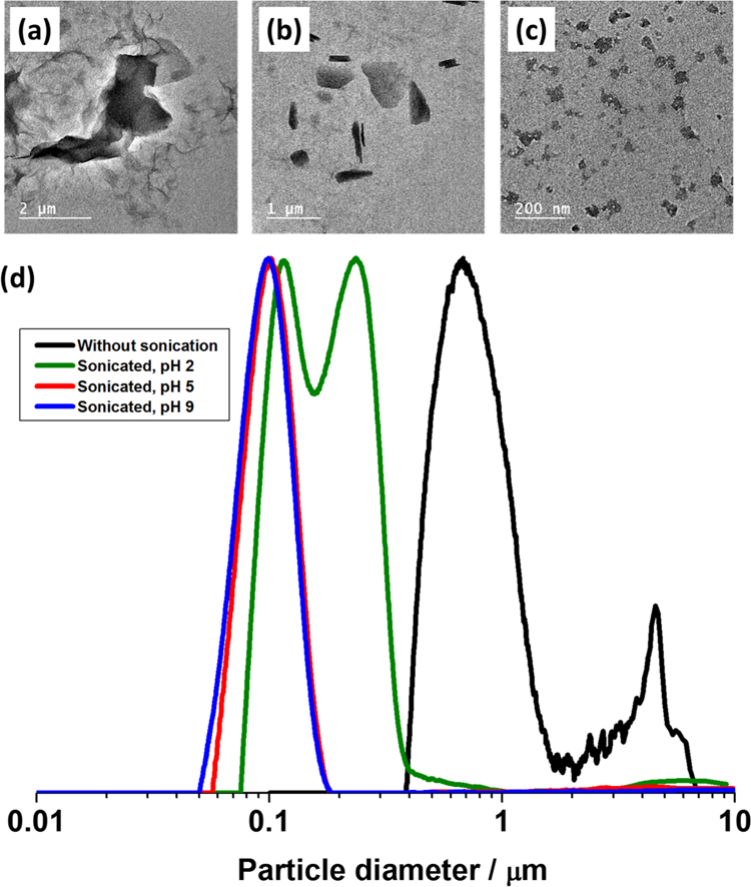
Representative TEM images of (a) commercial GO sheets as-received and GO nanosheets obtained after sonication at 70% amplitude for 30 min in aqueous solution at (b) pH 2 and (c) pH 5. (d) DCP particle size distributions obtained for corresponding GO sheets. The density of GO used in these measurements was taken as 1.2 g cm^–3^.

To further investigate the effect of solution pH, aqueous GO dispersions were diluted using water and adjusted to pH 2, 5, and 9 prior to sonication at 70% amplitude for 30 min. As expected, the size of the GO sheets became smaller after sonication than the original GO dispersion (Figure 2), and TEM studies indicated that the GO dispersed at higher pH resulted in smaller GO sheets after sonication (Figure 2b,c).

Disc centrifuge photosedimentometry (DCP) was also used to investigate the particle size distributions of the GO dispersions before and after sonication. DCP is a powerful technique to evaluate particle size distributions as it separates the particle population during analysis based on the size and relative density of the material. However, to provide accurate values for particle diameter, the technique assumes a spherical morphology and a single value for particle density.^54−56^ For GO, these assumptions are unlikely to be valid and, as such, the reported weight-average diameters should only be interpreted qualitatively. Nevertheless, for a given range of samples, the relative shape and position of the peaks reported by DCP can be used to interpret differences in particle diameter, number of particle populations, and degree of flocculation.

Figure 2d shows the particle size distributions for the commercial GO before and after sonication at pH 2, pH 5, and pH 9. Before sonication, the GO had a much broader size distribution, with particle sizes up to 7 μm, suggesting the GO was aggregated to some extent and not well dispersed during storage. In contrast, the peaks observed after sonication at pH 5 and pH 9 were clearly shifted from the nonsonicated peak. Furthermore, the particle size distributions were monomodal and narrower, with no evidence of flocculation. This indicated that well-dispersed GO sheets with smaller particle sizes were obtained. It is noteworthy that the peak of GO sonicated at pH 2 was still clearly shifted from nonsonicated peak, but the primary peak at approximately 0.1 μm was slightly shifted to a larger size, and a broader particle size distribution with two peaks was observed. This implies some degree of flocculation occurred after sonication. This can be attributed to a higher degree of protonation of the carboxyl groups at the GO sheet edges, resulting in lower charge repulsion. These observations are consistent with TEM studies (Figure 2a–c) and as such provide confidence in the use of DCP technique to analyze GO-containing dispersions.

### Control Heteroflocculation Experiments Using Anionic or Nonionic Latex Nanoparticles

Following the characterization of the polymer latexes and the GO dispersions, polymer/GO nanocomposite particles were prepared by heteroflocculation. Normally, there are four situations to consider for latex-GO mixtures: (i) the quantity of GO is insufficient to cover all of the surfaces latex particles; (ii) GO adsorbs onto the latex at monolayer coverage; (iii) GO is in excess, leading to the latex particles being fully coated, with excess GO either being present as a multilayer or remaining free in solution; and (iv) GO does not adsorb onto the latex, leading to the GO and latex coexisting in the dispersion.

**Figure 3:**
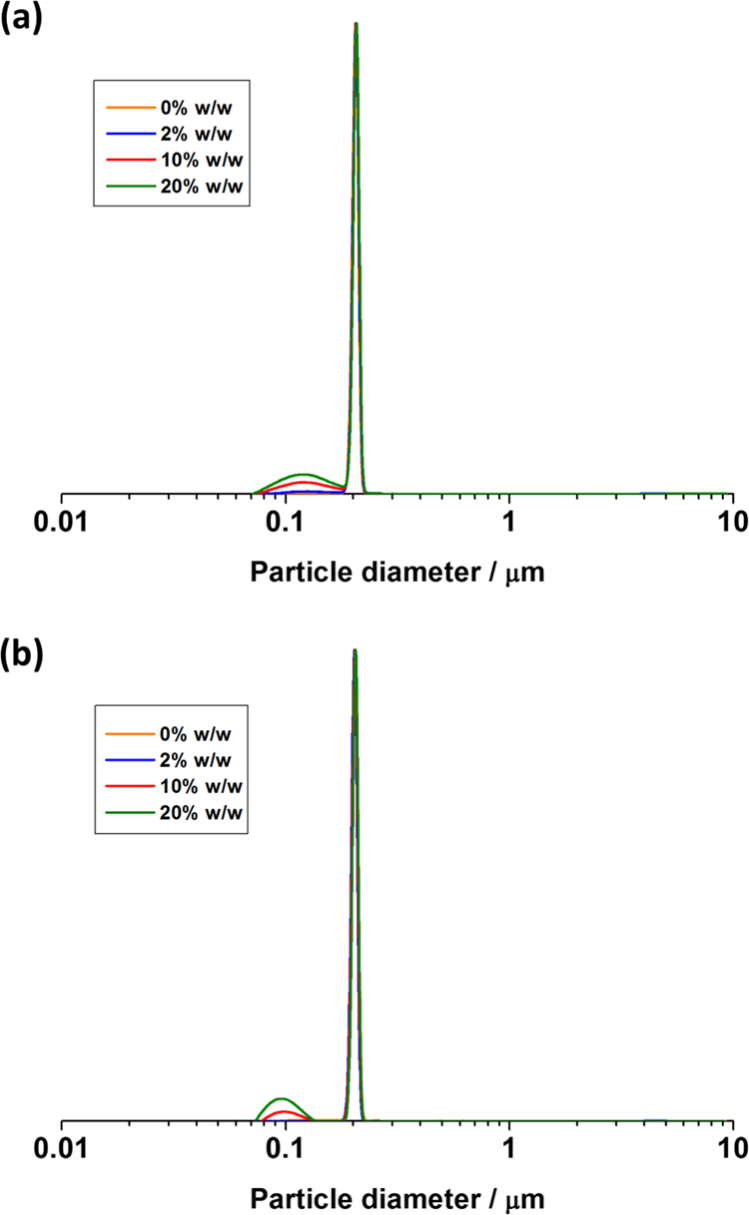
DCP particle size distributions obtained for anionic sterically stabilized PKSPMA_32_–PBzMA_300_ latex (entry 3, Table 1) before and after heteroflocculation with the addition of varying GO contents (2–20% w/w relative to latex) at (a) pH 2 and (b) pH 5. In both cases, GO did not adsorb onto the surface of the anionic particles, and thus the particle size traces of latexes were identical. The density used to calculate these particle size distributions was taken as 1.18 g cm^–3^.

Control heteroflocculation experiments were conducted to demonstrate the latter situation, where no adsorption was expected between negatively charged GO nanosheets and a negatively charged PKSPMA_32_–PBzMA_300_ latex (Figure 1c, entry 3 in Table 1).^38^Figure 3 shows DCP data for the anionic PKSPMA_32_–PBzMA_300_ latex before and after addition of up to 20% w/w GO at pH 2 and pH 5. A very narrow monomodal particle-size distribution was observed for the PKSPMA_32_–PBzMA_300_ latex. With the addition of GO nanosheets, no changes in the peak related to the polymer latex at approximately 0.2 μm were observed. However, broad shoulders at approximately 0.1 μm were observed, and the relative weight of this shoulder increased with increasing GO concentration. The small peak observed at approximately 0.1 μm is due to free GO and indicates that the latex particles and GO simply formed a binary mixture of noninteracting particles. Similar observations (see Figure S7) were recorded for heteroflocculation between GO and nonionic PBzMA latex (Figure 1d, entry 5 in Table 1).^39^

### Preparation of Polymer/GO Nanocomposite Particles *via* Heteroflocculation Using PEGMA-Stabilized P2VP Latex

Polymer/GO nanocomposite particles were prepared *via* heteroflocculation between cationic PEGMA-stabilized P2VP latex (PEGVP) and anionic GO nanosheets at room temperature (Scheme 1b). The PEGVP had a relatively high positive surface charge at pH 2 (ζ potential approximately +20 mV) and was relatively nonionic at pH 9 (ζ potential ∼0 mV, Figure S2a). In contrast, the GO was negatively charged (−20 to −40 mV) over a wide pH range (Figure S6) due to the presence of carboxylic acids on the sheet edges.^57^ Therefore, in this work, heteroflocculation between GO and the PEGVP latexes was investigated at pH 2, 5, and 9 by the addition of PEGVP latexes (0.1% w/w) to a stirred aqueous GO dispersion (0.1% w/w), see Table S2. The dispersions were mixed vigorously for 60 s and allowed to equilibrate using a roller mixer at room temperature for 48 h before analysis.

As the PEGVP latex was gradually added into the GO dispersion, aggregation was observed immediately, implying that the latex particles and GO sheets were interacting due to their opposite surface charges. Figure S8 shows digital photographs for various heteroflocculation experiments with varying GO contents using PEGVP latex (entry 4, Table 1). Upon standing, sedimentation occurred for most samples within 1 h, implying that the GO sheets were adsorbed onto the latex surfaces, causing bridging flocculation. At lower GO contents (<10% w/w, left two vials in Figure S8), most of the latex remained dispersed, but some sedimentation occurred, indicating the GO sheets were adsorbed onto the latex but were not providing colloidal stability. Furthermore, with increasing GO content, the dispersion color changed from white to transparent to dark. Darker coloration indicated that there was more free GO present in the dispersion. For PEGVP/GO prepared at pH 5 and pH 9, relatively transparent dispersions occurred at GO contents of 20% and 10% w/w (Figure S8b,c). However, at pH 2 (Figure S8a), transparent dispersions were observed at GO contents up to 100% w/w. This difference can be attributed to the PEGVP latexes being in their microgel form at pH 2 (Figure S2), resulting in a stronger electrostatic interaction and a larger surface area for GO adsorption.

The degree of flocculation of the polymer/GO nanocomposite particles can be assessed by comparing the DCP particle size distributions of latexes before and after the heteroflocculation process. Unfortunately, only one density can be used as an input in the DCP software for calculating the weight-average particle size, and thus only one accurate weight-average diameter can be determined per measurement. In the case of a binary particle mixture (latex + GO), this will inevitably lead to a relative error for one of the particle size distribution populations recorded. Furthermore, if heteroflocculation occurs, both the density and size of the original latex and resulting nanocomposite particles will necessarily be different, also leading to non-exact weight-average particle sizes. Nevertheless, if a single density value is used in all measurements, the relative positions/shifts of the peaks in the particle size distributions can be used to deduce whether heteroflocculation did occur to form individual nanocomposite particles and qualitatively assess the amount of free latex, free GO, and the degree of bridging flocculation.

**Figure 4:**
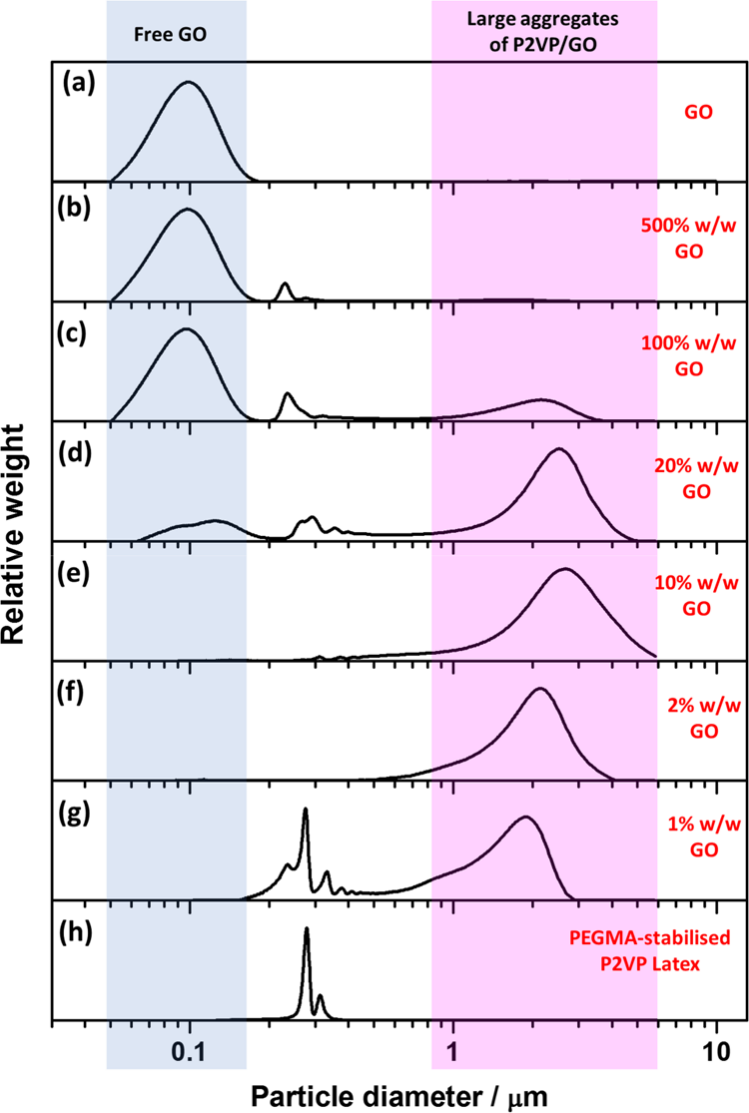
DCP particle size distributions obtained for PEGVP/GO nanocomposite particles prepared *via* heteroflocculation with varying GO contents at pH 9. The density used to calculate this data was taken as 1.11 g cm^–3^. Panel (a) represents GO nanosheets obtained *via* sonication at 70% amplitude for 30 min. Panels (b–g) represent entries 15–20 from Table S2, whereas panel (h) shows data obtained for entry 4 in Table 1.

Figure 4 shows DCP data for the PEGVP latex (entry 4, Table 1) before and after the addition of GO at pH 9. Figure 4h shows that the particle size distribution of the PEGVP latex was relatively narrow, with a small shoulder attributed to a small population of dimers. With the addition of GO nanosheets, the narrow particle size distribution of the PEGVP latex becomes broader, and the mean particle diameter significantly increases, indicating the formation of polymer/GO nanocomposite particles. Similar observations have been previously reported for PEGVP/titania nanocomposite particles.^36^Figure 4g shows that significant flocculation of PEGVP latexes occurred, and only a small amount of free latex was observed even after only adding 1% w/w GO based on latex. This indicated that the GO strongly adsorbed onto the latexes but caused bridging flocculation. With increasing GO contents (Figure 4b–f), no free PEGVP latexes were observed, and large polymer/GO nanocomposite aggregates formed. Furthermore, with GO contents higher than 20% w/w (Figure 4b–d), the free GO peak at 0.1 μm is more obvious, indicating that the latex particles were fully covered by GO and free GO nanosheets were dispersed in the solution. In contrast, only a small free GO peak was observed at 10% w/w (Figure 4e), indicating that the GO content may be below or equal to the amount GO needed to cover the surface of the latex particles present. Therefore, the optimal quantity of GO for the 222 nm PEGVP latex was between 10 and 20% w/w.

**Figure 5:**
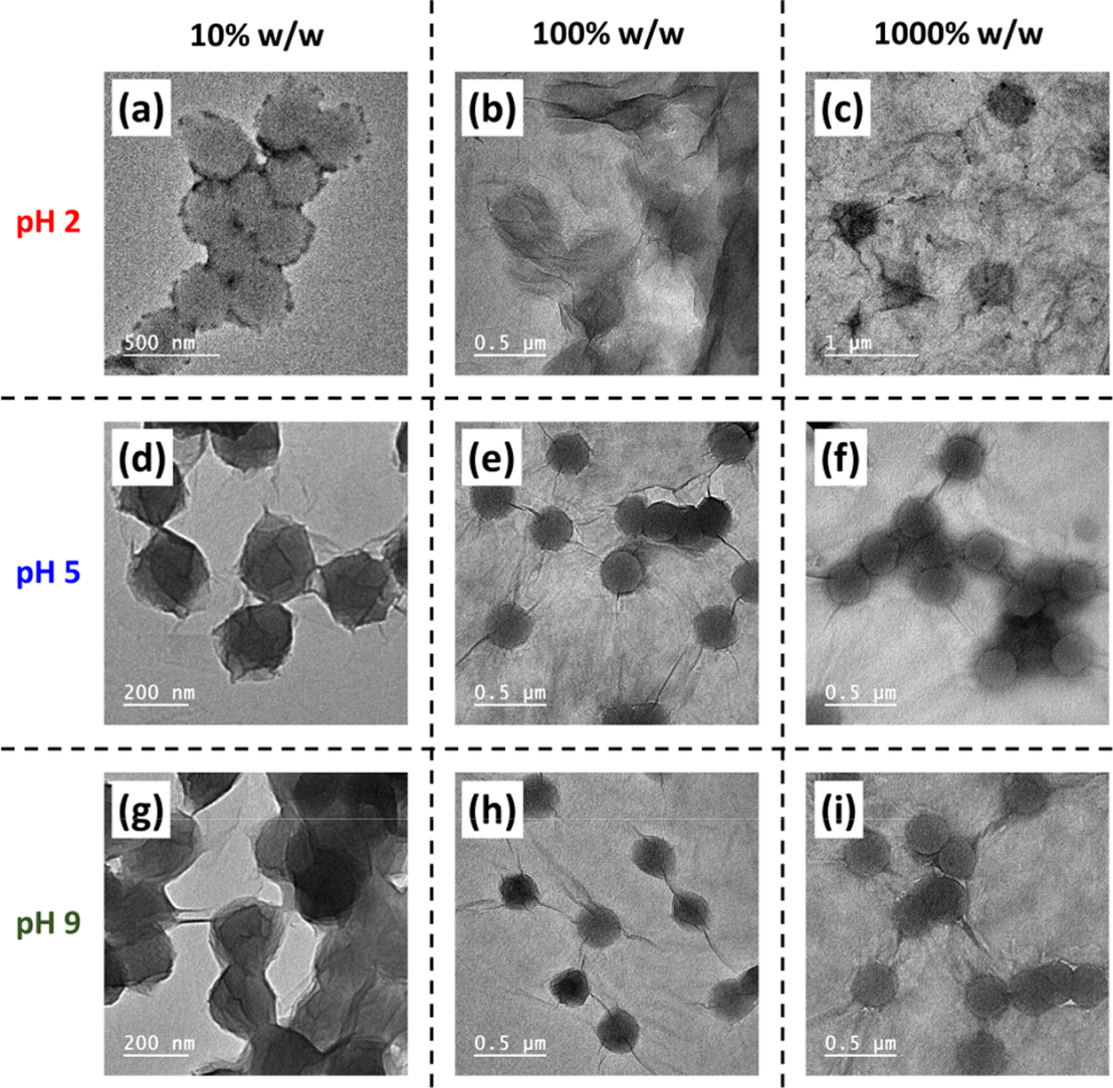
Representative TEM images for polymer/GO nanocomposite particles prepared *via* heteroflocculation between PEGVP latex and GO with varying GO contents (10%, 100%, and 1000% w/w). Images (a–c), (d–f) and (g–i) correspond to heteroflocculation conducted in aqueous solution at pH 2, 5, and 9, respectively (entries 3, 5, 7, 10, 12, 14, 17, 19, and 21; Table S2).

It is noteworthy that small peaks with a similar size to the PEGVP latexes were observed at GO contents higher than 20% w/w (Figure 4b–d). These small peaks can be ascribed to a population of nonaggregated individual PEGVP/GO nanocomposite particles (Figure 5g). As mentioned above, only one density can be used as an input in the DCP software for calculating the weight-average particle size. As the GO would be very hydrated, the individual PEGVP/GO nanocomposite particles would have a lower effective density than pristine PEGVP latexes,^58^ and therefore the particle size determined by the DCP software may not accurately reflect the actual particle diameter.^59^

Figure 5 shows representative TEM images for the PEGVP/GO nanocomposite particles prepared *via* heteroflocculation with varying GO contents at pH 2, 5, and 9. Nanocomposite particles were obtained at pH 5 and pH 9 with a GO content of 10% w/w (Figure 5d,g). This indicated that even though the latexes had a relatively low surface charge at pH 9, the GO could still adsorb onto the latexes *via* electrostatic interactions. It is noteworthy that there was no free GO observed on the TEM grids, and the latexes appeared to be fully coated with GO nanosheets. When using higher GO contents, the latexes appeared to simply imbed or load onto the GO sheets.

It is noteworthy that the ζ potential values of nanocomposite particles were generally between the ζ potential value of the pristine GO and PEGVP latex nanoparticles (Table S2). Furthermore, when comparing the samples with the same GO content, the nanocomposite particles prepared at higher solution pH had a higher surface charge. For example, the ζ potential of PEGVP/GO prepared using 10% w/w GO at pH 5 and pH 9 was −11 and −29 mV, respectively. This can be attributed to the adsorbed GO sheets on the surface of the latexes being more negatively charged at higher pH.

At pH 2, the observed morphology was not well-defined even at a GO content of 10% w/w (Figure 5a, entry 3 in Table S2). This may be attributed to the GO having a relatively low negative charge at pH 2, leading to aggregation of the GO sheets (Figure 2). Alternatively, the microgel form of these latexes at this pH may also hinder the observation of these particles when dried and observed under the high-vacuum conditions of an electron microscope. Overall, for the preparation of PEGVP/GO nanocomposite particles, the relatively optimal conditionals can be considered to be a solution pH of 5 to 9 with a GO content between 10 and 20% w/w.

### Preparation of Polymer/GO Nanocomposite Particles *via* Heteroflocculation Using P2VP–PBzMA Latexes

Polymer/GO nanocomposite particles were prepared *via* heteroflocculation between cationic sterically stabilized V_x_-B_300_ latex nanoparticles and GO nanosheets (Scheme 1c). Similar to the PEGVP latex discussed above, the V_x_-B_300_ latexes were pH-responsive. More specifically, the latexes are highly positively charged at low pH (Figures S3a and S4a) due to the higher degree of protonation of the pyridine groups on the P2VP stabilizer. Therefore, electrostatic interactions between positively charged V_x_-B_300_ and negatively charged GO sheets is probable. This also implied that polymer/GO nanocomposite particles can be potentially prepared *via* electrostatically induced heteroflocculation using block copolymer nanoparticles, with P2VP as the stabilizer.

As the latex was gradually added into the GO dispersions, coagulation was observed immediately, indicating that the latex particles and GO sheets were associating due to the oppositely charged surfaces. Figures S9 and S10 show similar appearances for both V_32_–B_300_/GO and V_67_–B_300_/GO nanocomposite particles prepared *via* heteroflocculation at pH 2, 5, and 9. This was generally consistent with the heteroflocculation between PEGVP and GO (Figure S8) as follows: (i) upon standing, sedimentation occurred within 1 h; (ii) at lower GO content (1% and 2% w/w, left two vials), most of the latexes still remained dispersed, but sedimentation occurred due to bridging flocculation; (iii) relatively clear dispersions were obtained with the GO content of 20% and 10% w/w at pH 5 and pH 9; and (iv) at pH 2, clear dispersions were observed at GO contents up to 100% w/w.

**Figure 6:**
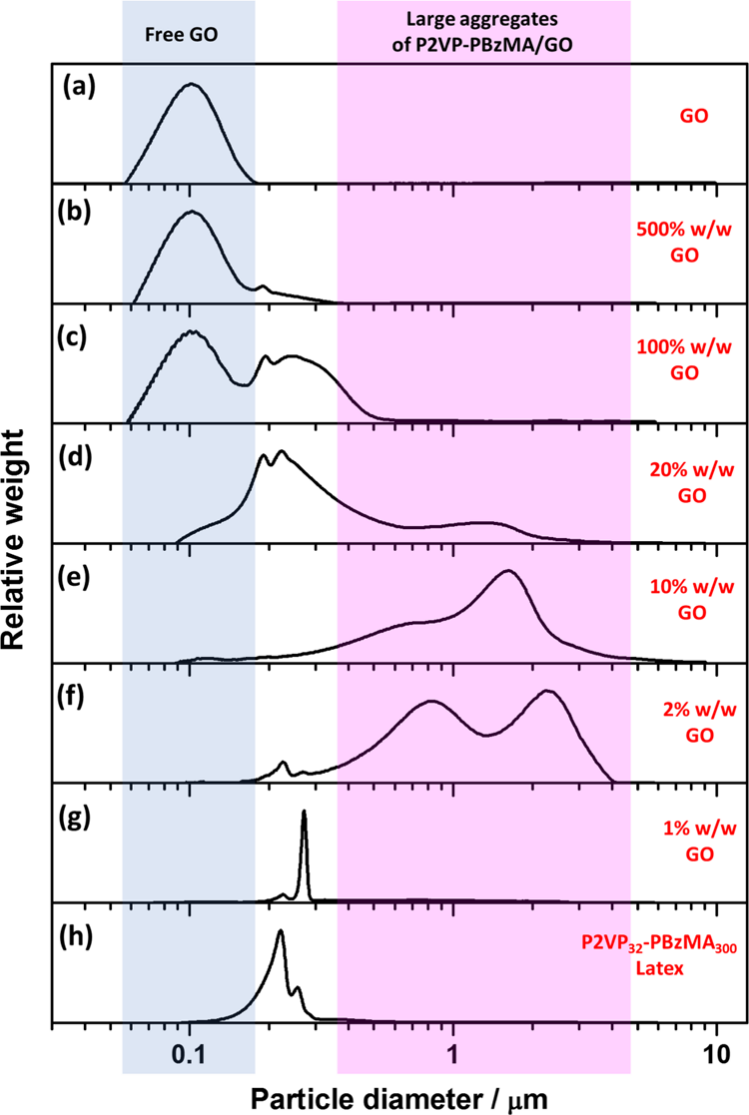
DCP particle size distributions obtained for V_32_-B_300_/GO nanocomposite particles prepared *via* heteroflocculation with varying GO contents at pH 5. The density of the latex and nanocomposite particles was taken as 1.18 g cm^–3^. Panel (a) Represents GO nanosheets obtained *via* sonication at 70% amplitude for 30 min. Panels (b–g) represent entries 8–13 from Table S3, whereas panel (h) shows data obtained for entry 1 in Table 1.

Figure 6 shows DCP data obtained for V_32_-B_300_ latex nanoparticles (entry 1, Table 1) before and after the addition of GO at pH 5. The particle size distribution obtained for the bare latex was relatively narrow (Figure 6h). Similar to the observation for the electrostatically induced heteroflocculation between PEGVP latexes and GO, with the addition of GO nanosheets, the narrow particle size distribution of the V_32_-B_300_ latex became broader and the mean weight-average particle diameter increased. At GO contents higher than 2% w/w (Figure 6b–f), the particle size distribution became much broader than that of the primary latex, indicating that the V_32_-B_300_/GO nanocomposite aggregates were formed. When using 10% w/w GO (Figure 6e), large aggregates are still observable and there is little evidence for any free latex or individual polymer/GO particles. However, obvious peaks of individual V_32_-B_300_/GO nanocomposite particles were observed at GO content higher than 20% w/w (Figure 6b–d). Similar observations were made for the V_32_–B_300_/GO (Figure S11) and V_67_–B_300_/GO (Figure S12) nanocomposite particles obtained *via* heteroflocculation at pH 2. However, GO nanosheets are aggregated to some extent at pH 2 due to a relatively low negative charge, making it relatively difficult to clearly distinguish bridging flocculation from individual nanocomposite particles and free GO. Nevertheless, the broad DCP distributions and TEM studies (Figures S13 and S14) suggested that the P2VP–PBzMA/GO nanocomposite particles were obtained in both cases.

Morphologies of V_32_-B_300_/GO and V_67_-B_300_/GO nanocomposite particles prepared *via* heteroflocculation at pH 2, 5, and 9 are shown in Figures S13 and S14, respectively, and appear relatively similar to the PEGVP/GO nanocomposites in Figure 5. In addition, the ζ potential values of the nanocomposite particles were generally between the ζ potential value of the GO and P2VP–PBzMA latex nanoparticles (Tables S3 and S4). Similar to PEGVP/GO (Figure 5a), the V_x_-B_300_/GO nanocomposite particles prepared using 10% w/w GO at pH 2 were not well-defined (Figures S13a and S14a). As previously discussed, this may be attributed to the GO having a relatively low negative charge at pH 2, leading to aggregation among GO sheets (Figure 2). Therefore, only a small amount of GO can be adsorbed as sheets on the latex surface.

### Determination of Free GO after Heteroflocculation Using UV–vis Spectrophotometry

Figure S15a shows UV–vis absorbance spectra of GO aqueous dispersions at varying concentrations ranging from 0.001 to 0.067 mg mL^–1^. The spectra indicated that the GO is a strong UV absorber, and the wavelength of the maximum absorption peak was approximately 235 nm, which is consistent to previously reported values.^47^Figure S15b shows a linear relationship between the absorbance at 235 nm and the GO concentration. This indicated that the GO dispersions obey the Beer–Lambert law, with the coefficient of absorbance (ε) calculated to be 47 mL mg^–1^ cm^–1^. Therefore, the mass fraction of free GO nanosheets after the heteroflocculation process could be determined using UV–vis spectroscopy.^47^ More specifically, the heteroflocculation dispersions were centrifuged at moderate speed (200 rpm) for 5 min. At this low speed, the polymer/GO nanocomposite particles would sediment, and the free GO nanosheets remained dispersed in the supernatant. The supernatants were carefully collected and diluted using water at the corresponding pH. The diluted supernatants were analyzed *via* UV–vis spectroscopy to determine the quantity of free GO not absorbed onto the latexes.

**Figure 7:**
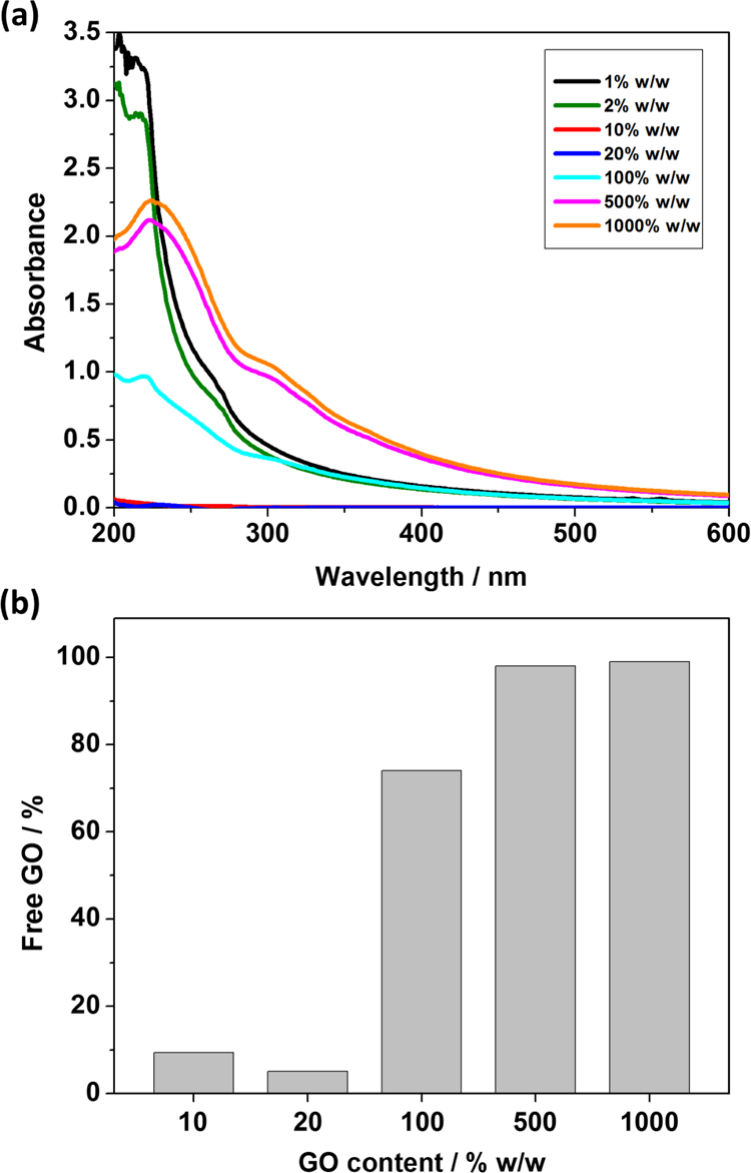
(a) UV–vis spectra for diluted supernatants obtained from centrifuged heteroflocculation samples prepared using V_32_–B_300_ latexes with varying GO contents at pH 5 (entries 8–14, Table S3). (b) Calculated mass fraction of free GO for corresponding heteroflocculation samples.

Figure 7a shows UV–vis spectra of the diluted supernatant obtained from dispersions of V_32_–B_300_ latex mixed with varying amounts of GO at pH 5 (entries 8–14, Table S3). Strong UV signals at approximately 220 nm (corresponding to latex) were observed, especially for the formulations using lower GO content (1% and 2% w/w). In contrast, stronger UV signals at 235 nm (from GO) were observed for the formulations using higher GO contents, with peaks due to latex becoming negligible. It is noteworthy that UV signals of both the latex and GO were negligible at the GO content of 10% and 20% w/w, indicating only limited free latex and free GO remained dispersed in the supernatants. This observation is consistent with the digital photographs, DCP analysis, and TEM images discussed above.

For V_32_–B_300_/GO samples with UV signals of free GO, the lowest mass fraction of free GO was approximately 5% for the formulation using 20% w/w GO (Figure 7b and entry 11 in Table S3). This indicated that most of the GO nanosheets were adsorbed on the latex surfaces, and only limited residual GO is still dispersed in the solution. This also implied that V_32_–B_300_/GO nanocomposite particles with monolayer coverage were achieved at the GO content of approximately 20% w/w.

### Investigating the Electrostatic Interaction Strength between Latex and GO Nanosheets after Heteroflocculation

The strength of electrostatic interaction between latex nanoparticles and GO nanosheets was assessed by comparing the DCP particle size distributions before and after a short period of sonication in a bath. More specifically, dispersions prepared using GO contents of 10% and 20% w/w, based on latex, were sonicated using an 820 W sonicator bath at a fixed frequency of 37 kHz for 60 s and subsequently analyzed *via* DCP.

**Figure 8:**
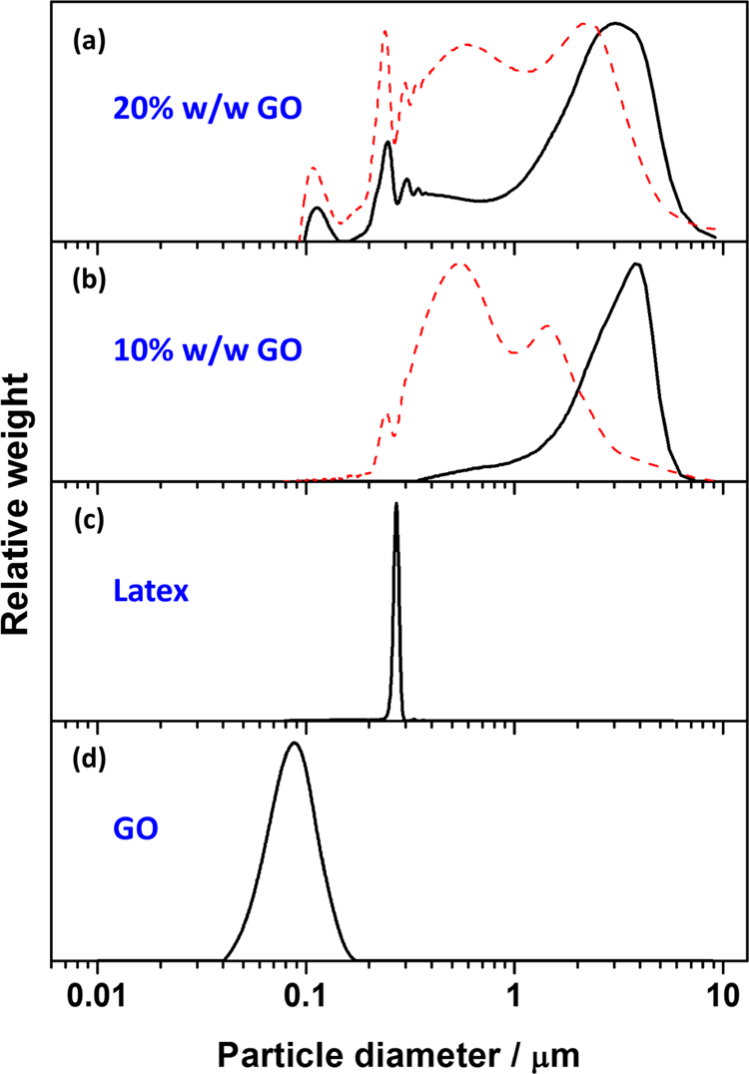
DCP particle size distributions obtained before (solid line) and after (dashed line) sonication for PEGVP/GO nanocomposite particles prepared at pH 5 with GO contents of (a) 20% w/w (entry 11, Table S2) and (b) 10% w/w (entry 10, Table S2), (c) PEGVP latex (entry 4, Table 1), and (d) GO nanosheets obtained *via* sonication at 70% amplitude for 30 min in aqueous solution at pH 5. For ease of comparison, the density used for all DCP analyses was fixed as 1.11 g cm^–3^.

Figure 8 shows DCP particle-size distributions obtained for PEGVP/GO nanocomposite particles before (solid line) and after (dashed line) sonication. After sonication, the distributions were still broader than the primary latex peak. However, the distributions obviously shifted to a smaller size after sonication, indicating that the aggregates significantly decreased in size, and some individual PEGVP/GO nanocomposite particle peaks (at approximately 0.25 μm) were observed (Figure 8a,b). This implied that the bridging flocculation of PEGVP/GO nanocomposite particles can be disrupted using sonication. It is noteworthy that the PEGVP latex was stabilized by nonionic PEGMA stabilizer, which can screen the positive charge generated by cationic P2VP core. This led to the electrostatic interaction between PEGVP latex and GO nanosheet being relatively weak. Therefore, it is likely that some GO coated on the PEGVP latexes becomes detached during sonication, for example, a small free GO peak (at approximately 0.1 μm) becomes apparent after sonication of PEGVP/GO (Figure 8a).

**Figure 9:**
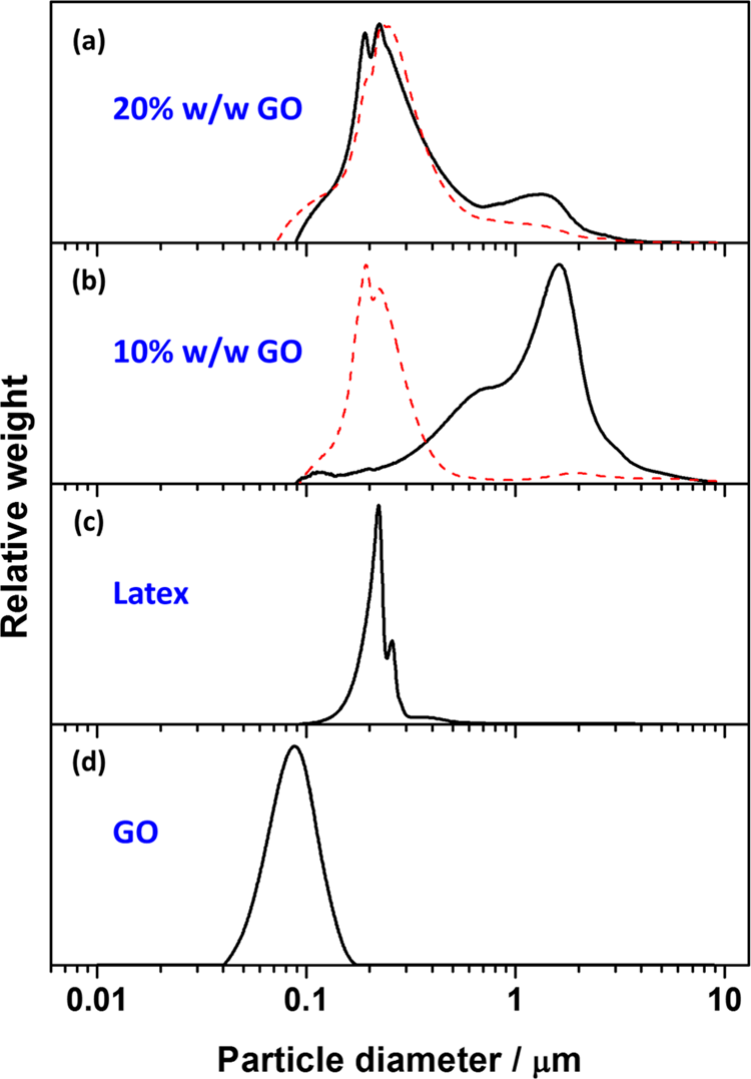
DCP particle size distributions obtained before (solid line) and after (dashed line) sonication for V_32_–B_300_/GO nanocomposite particles prepared at pH 5 with GO contents of (a) 20% w/w (entry 11, Table S3), (b) 10% w/w (entry 10, Table S3), (c) V_32_–B_300_ latex (entry 1, Table 1), and (d) GO nanosheets obtained *via* sonication at 70% amplitude for 30 min in aqueous solution at pH 5. For ease of comparison, the density used for all DCP analyses was fixed as 1.18 g cm^–3^.

Similar observations were made for V_32_-B_300_/GO (Figure 9) and V_67_–B_300_/GO (Figure S16) nanocomposite particles before (solid line) and after (dashed line) sonication. At a GO content of 10% w/w, the particle size decreased significantly and the individual V_x_-B_300_/GO nanocomposite particles’ peaks (at approximately 0.2 μm) were observed. This indicated that the bridging flocculation of these nanocomposite particles can also be disrupted by sonication. It is noteworthy that a relatively minor particle-size distribution change was observed for the V_x_-B_300_/GO nanocomposite particles prepared using 20% w/w GO after sonication (Figures 9a and S16a). This implied that most of the V_x_-B_300_/GO nanocomposite particles generally remained the same size as individual nanocomposite particles. Furthermore, compared to PEGVP/GO, a relatively small amount of free GO nanosheets (at approximately 0.1 μm) were generated, implying that only a limited quantity of coated GO was detached from the surface of the V_x_-B_300_ latexes after sonication. This indicated that the electrostatic interaction between V_x_-B_300_ latexes and GO nanosheet was stronger than that between PEGMA-stabilized P2VP latex and GO nanosheets.

## Conclusions

Polymer/GO nanocomposite particles were prepared *via* electrostatically induced heteroflocculation in an aqueous medium at room temperature using positively charged latex nanoparticles and negatively charged GO nanosheets. DCP studies indicated that polymer/GO nanocomposite particles were successfully formed either using PEGMA-stabilized P2VP or P2VP–PBzMA latexes, and the optimal GO loading was approximately 20% w/w based on latex. This was consistent with the calculated mass fraction of free GO determined *via* UV–vis analysis. TEM studies confirmed that the GO nanosheets were adsorbed on the latex surface, especially at pH 5 and pH 9. Aqueous electrophoresis showed that the ζ potential values of nanocomposite particles were generally between the ζ potential values of the GO and latex nanoparticles. Furthermore, when comparing the samples with the same GO content, the nanocomposite particles prepared at higher solution pH had a higher surface charge, indicating that the GO sheets were adsorbed on the surface of the latexes. Control heteroflocculation experiments conducted using anionic and latexes resulted in no polymer/GO nanocomposite particles being formed.

The strength of electrostatic interaction between cationic latex nanoparticles and GO nanosheets was assessed by comparing the DCP particle size distributions before and after sonication. The particle size distributions obviously shifted to a smaller size after sonication, indicating the occurrence of bridging flocculation, which could readily be disrupted. Furthermore, a smaller amount of free GO nanosheets were generated after sonication of P2VP–PBzMA/GO than PEGVP/GO nanocomposite particles, indicating that the electrostatic interaction between P2VP–PBzMA latex and GO was stronger than that between PEGMA-stabilized P2VP and GO.

The preparation of polymer/GO nanocomposite particles reported herein is a promising approach toward the formation of functional two-dimensional (2D) material/polymer nanocomposites, and thus this generic approach could be substantially extended to other colloidal polymer/2D nanoparticle combinations.

## Supporting Information Available

Latex particle-size distributions and aqueous electrophoresis studies; a schematic representation of GO; summary of GO particle sizes obtained after ultrasonication; aqueous electrophoresis studies of GO dispersion; DCP particle-size distributions obtained for nonionic PBzMA latex before and after heteroflocculation with GO; summary tables, digital photographs and DCP particle-size distributions for heteroflocculation studies between GO and latexes; representative TEM images for polymer V_67_–B_300_/GO nanocomposite particles; UV–vis spectra and Beer–Lambert plot obtained for GO aqueous dispersions; and DCP particle-size distributions obtained before and after sonication for V_67_–B_300_/GO nanocomposite particles. This material is available free of charge *via* the Internet at http://pubs.acs.org (PDF)
