# Supplementary material for: Physical Adsorption of Graphene Oxide onto Polymer Latexes and Characterization of the Resulting Nanocomposite Particles
Source: Langmuir. 2022 Jun 30;38(27):8187–99. doi: 10.1021/acs.langmuir.2c00327 (PMC9281389; doi:10.1021/acs.langmuir.2c00327)
Supplement: Supplementary file 1 — la2c00327_si_001.pdf [file la2c00327_si_001.pdf]

Supporting Information

Physical adsorption of graphene oxide onto polymer latexes and characterization of the resulting nanocomposite particles

Shang-Pin Wen,[a] Elisabeth Trinh,[a] Qi Yue [a] and Lee A. Fielding[a, b]*

[a] Department of Materials, School of Natural Sciences, University of Manchester, Oxford Road, Manchester, M13 9PL, UK. E-mail: lee.fielding@manchester.ac.uk

[b] Henry Royce Institute, The University of Manchester, Oxford Road, Manchester, M13 9PL, UK.

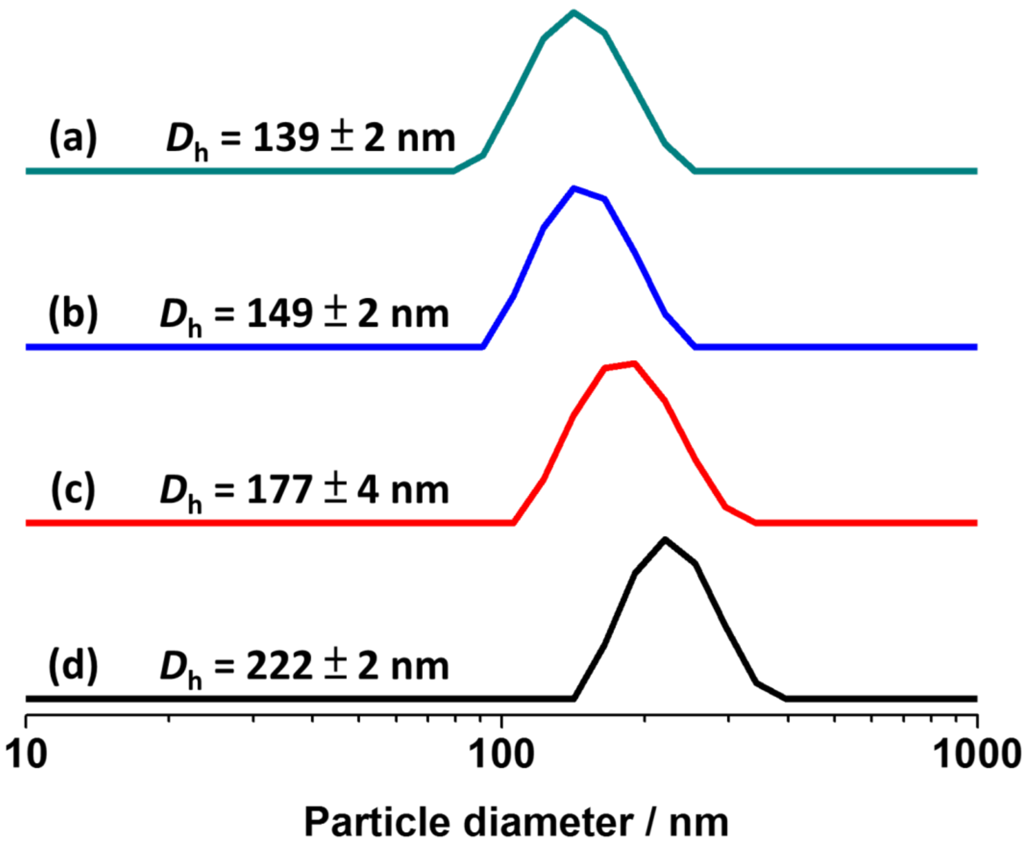

Figure S1. Intensity size distributions obtained *via* DLS for (a) $P2VP_{32}$–$PBzMA_{300}$, (b) $P2VP_{67}$–$PBzMA_{300}$, (c) $PKSPMA_{32}$–$PBzMA_{300}$ and (d) PEGMA-stabilized P2VP latexes. Traces (a), (b), (c) and (d) represent entries 1–4 in Table 1, respectively.

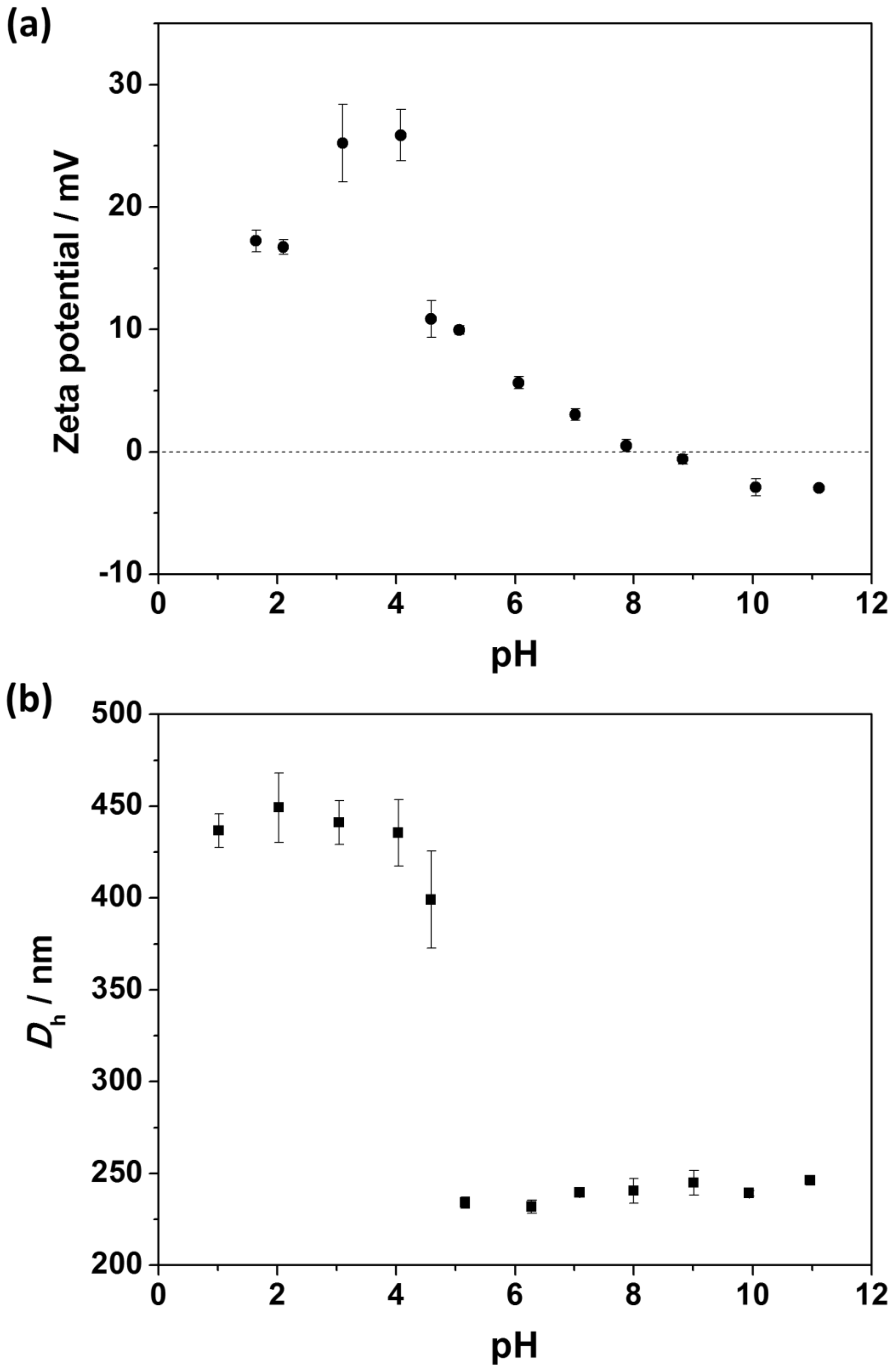

Figure S2. (a) Zeta potential and (b) mean hydrodynamic diameter (D_h) as a function of pH obtained for PEGVP latex (entry 4, Table 1). Measurements were conducted at a latex concentration of approximately 0.1 % w/w with 1 mM KCl as a background electrolyte. The solution pH was initially adjusted to pH 11 by the addition of KOH and subsequently lowed to pH 1.0 using HCl.

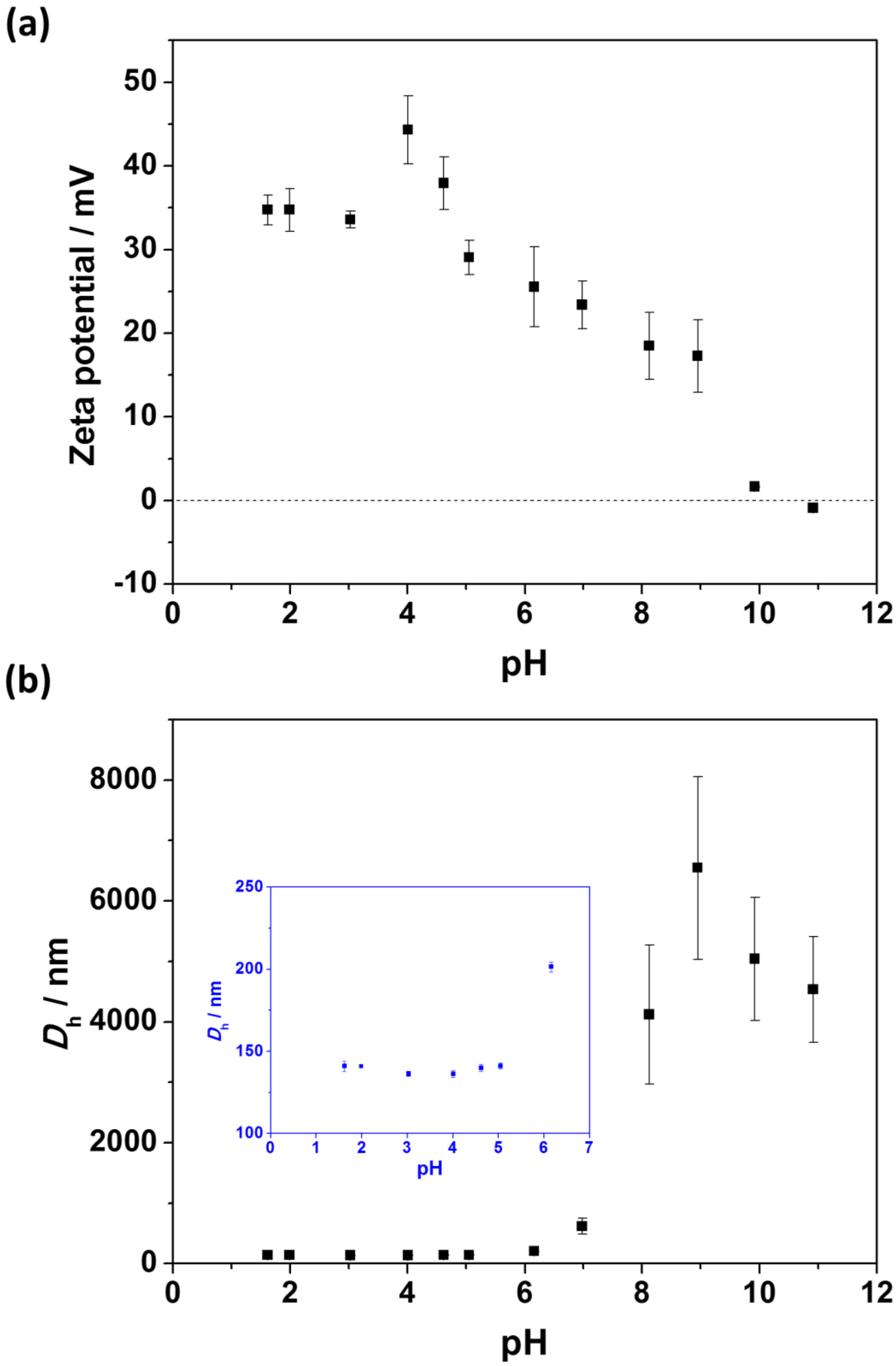

Figure S3. (a) Zeta potential and (b) mean hydrodynamic diameter (D_h) as a function of pH obtained for V_{32}–B_{300} latex (entry 1, Table 1). Measurements were conducted at a latex concentration of approximately 0.1% w/w with 1 mM KCl as a background electrolyte. The solution pH was initially adjusted to pH 1.5 by the addition of HCl and subsequently titrated to pH 11 using KOH. The inset shows the magnification of the particle diameter changes below pH 7.

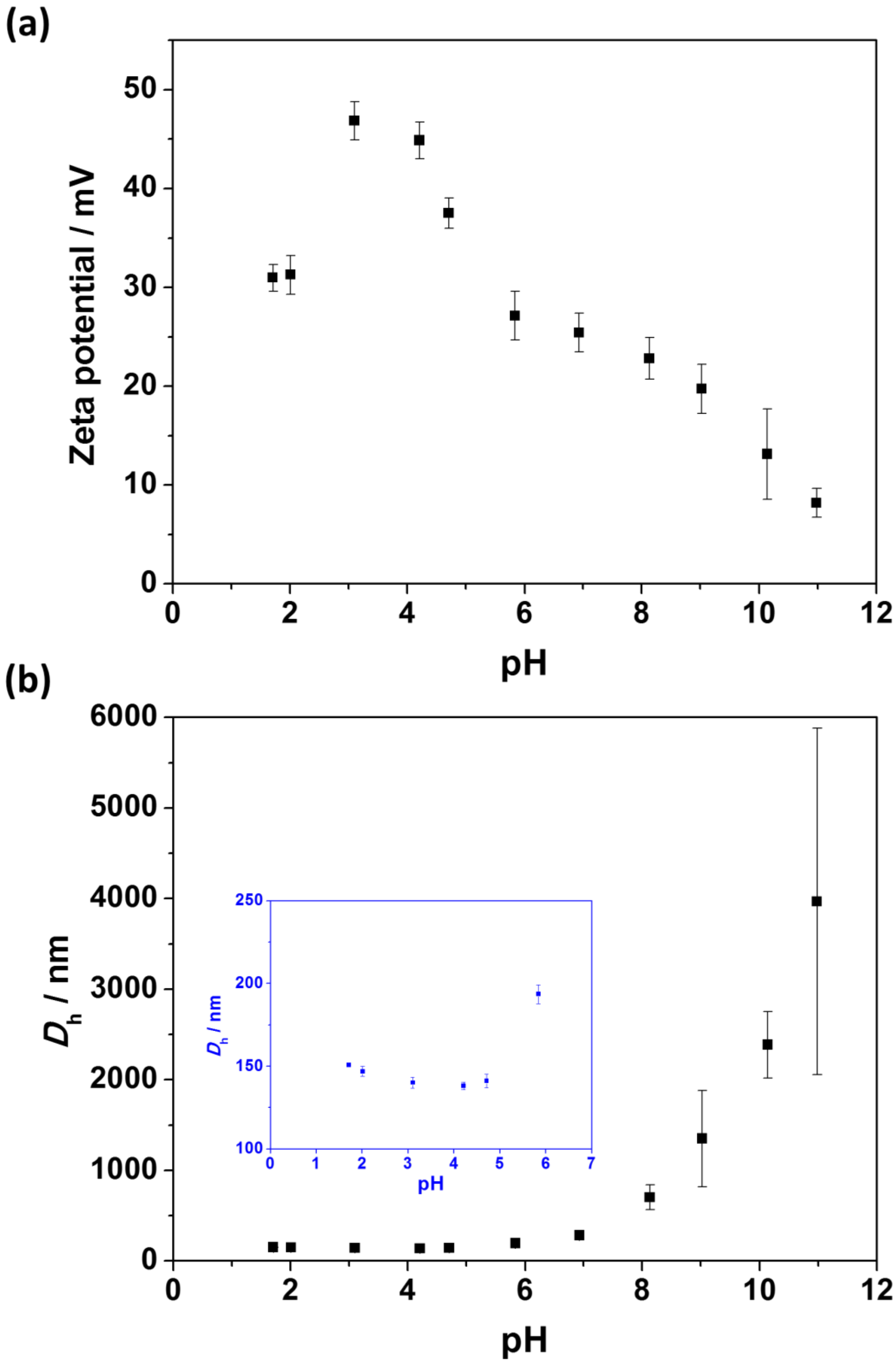

Figure S4. (a) Zeta potential and (b) mean hydrodynamic diameter (D_h) as a function of pH obtained for V_{67}–B_{300} latex (entry 2, Table 1). Measurements were conducted at a latex concentration of approximately 0.1% w/w with 1 mM KCl as a background electrolyte. The solution pH was initially adjusted to pH 1.5 by the addition of HCl and subsequently titrated to pH 11 using KOH. The inset shows the magnification of the particle diameter changes below pH 7.

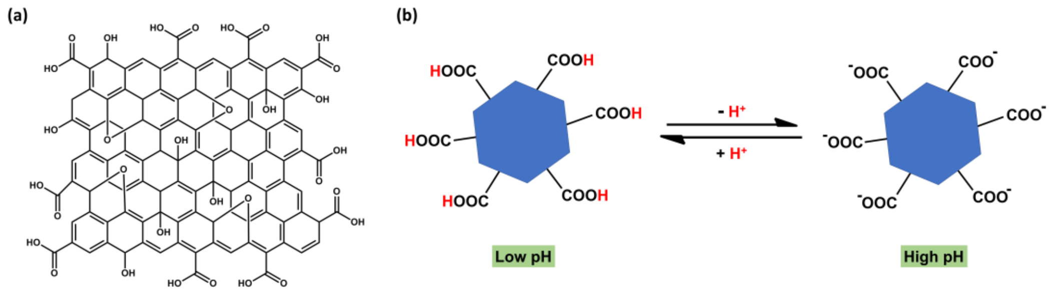

Scheme S1. (a) Two-dimensional GO structure with hydroxyl (–OH) and epoxy groups on the basal plane and carboxyl (–COOH) groups on the edges.[1, 2] (b) The –COOH groups on the edge of GO can be reversibly protonated at different pH.

Table S1. Summary of GO dispersions obtained using ultrasonication with varying parameters.

Entry	Amplitude / %	Process time / min	$D_{h, To}$[b] / nm	$D_{h, 3D}$[c] / nm
1[a]			1448 ± 120	
2	70	5	395 ± 8	447 ± 6
3	70	10	341 ± 5	391 ± 11
4	70	30	235 ± 2	286 ± 2
5	90	5	425 ± 13	470 ± 23
6	90	10	375 ± 6	403 ± 2

[a] Commercial GO dispersion received from Graphenea (Spain) without sonication.

[b] Obtained *via* DLS immediately after sonication.

[c] Obtained *via* DLS after storage for 3 days. The GO concentration was fixed at 4 mg mL^{-1}.

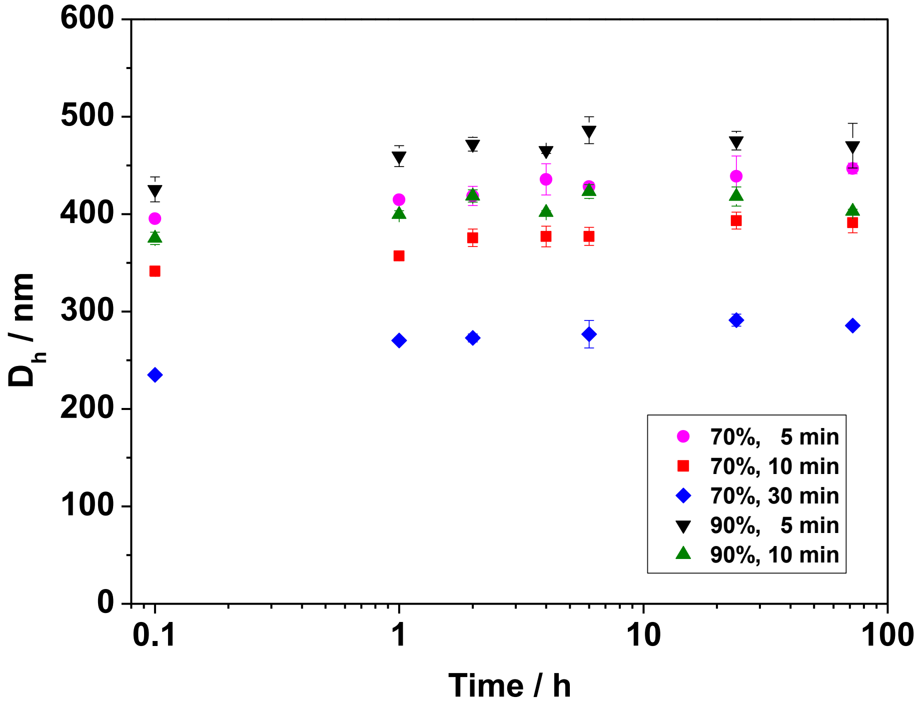

Figure S5. Mean hydrodynamic diameter (D_h) of GO sheets obtained after ultrasonication using various parameters [amplitude (70% or 90%) and process time (5, 10 or 30 min)] and over the course of being monitored for 3 days. The GO concentration was fixed at 4 mg mL^{-1}.

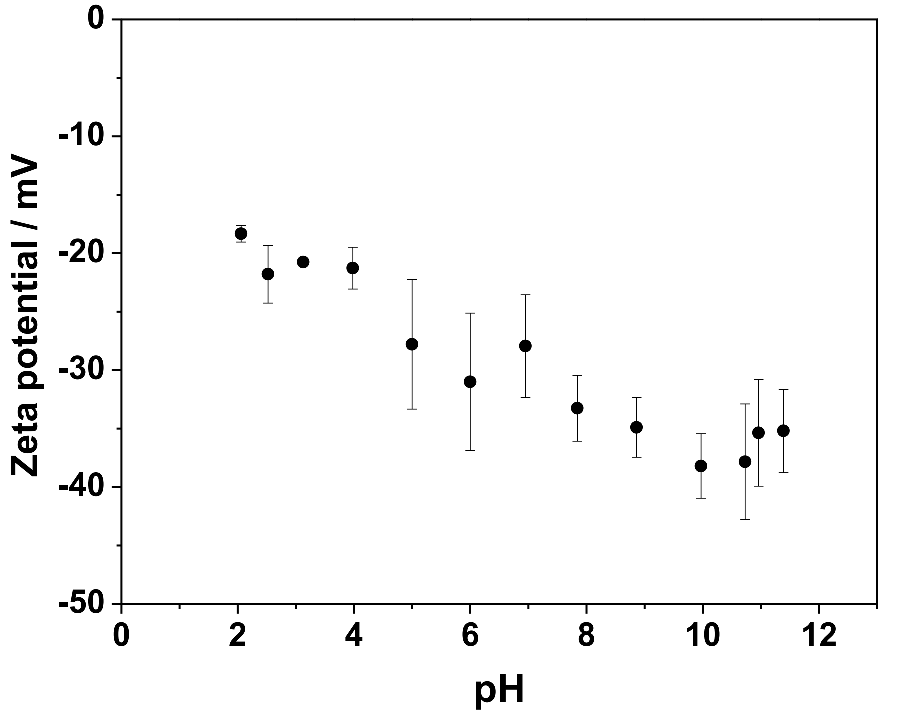

Figure S6. Aqueous electrophoresis data as a function of pH obtained for a GO dispersion (entry 4, Table S1) at a concentration of approximately 0.1% w/w in the presence of 1 mM KCl as a background electrolyte. The solution pH was initially adjusted to pH 2 by the addition of HCl and subsequently titrated to pH 12 using KOH.

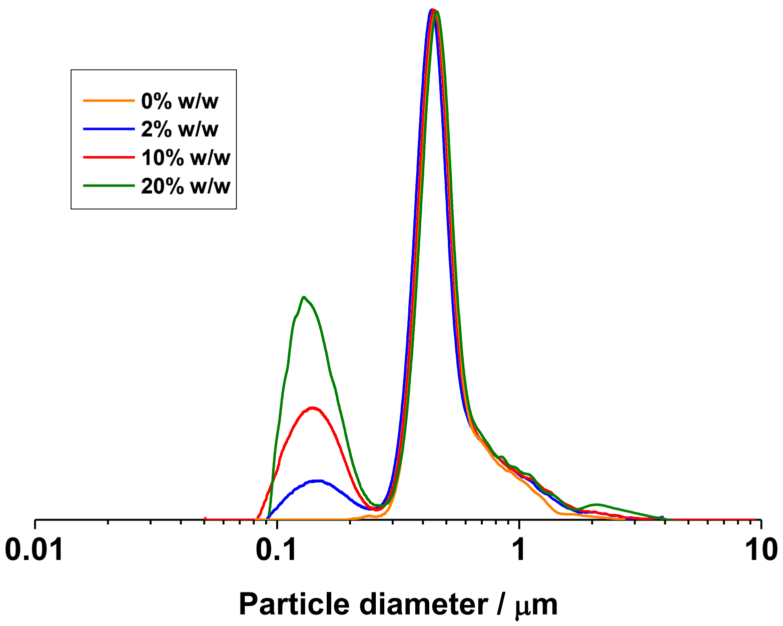

Figure S7. DCP particle size distributions obtained for non-ionic PBzMA latex nanoparticles[3] (D_h = 311 nm) before and after heteroflocculation with addition of varying GO content (2 to 20 % w/w relative to latex) at pH 9. In all cases, the anionic GO was not adsorbed onto the surface of the non-ionic PBzMA particles, and thus the particle size traces of latexes were identical. The density used to calculate these particle size distributions was taken as 1.18 g cm^{-3}.

Table S2. Summary of polymer/GO nanocomposite particles prepared *via* heteroflocculation between 222 nm PEGVP latex and GO nanosheets at varying pH in dilute aqueous solution at room temperature. The solids content was fixed at 0.1 % w/w.

Entry	Solution pH	$D_{h,\ GO}$ [a] / nm	GO content [b] / % w/w	Free GO [c] / %	Zeta potential [d] / mV
1	2	292	1		+17 ± 1
2	2	292	2		+18 ± 1
3	2	292	10		+16 ± 1
4	2	292	20	21	+17 ± 1
5	2	292	100	2	-10 ± 1
6	2	292	500	82	-21 ± 0
7	2	292	1000	75	-20 ± 1
8	5	111	1		+21 ± 2
9	5	111	2		+2 ± 1
10	5	111	10	2	-11 ± 4
11	5	111	20	36	-29 ± 0
12	5	111	100	88	-30 ± 1
13	5	111	500	>99	-37 ± 0
14	5	111	1000	>99	-38 ± 2
15	9	103	1		-6 ± 1
16	9	103	2		-3 ± 4
17	9	103	10	10	-29 ± 0
18	9	103	20	73	-32 ± 0
19	9	103	100	>99	-34 ± 1
20	9	103	500	>99	-38 ± 0
21	9	103	1000	>99	-40 ± 1

[a] Mean hydrodynamic diameter of GO determined *via* DLS analysis.

[b] Concentration relative to latex.

[c] Mass fraction of free GO relative to GO added, as determined by UV-Vis analysis.

[d] Determined *via* aqueous electrophoresis analysis at the corresponding solution pH.

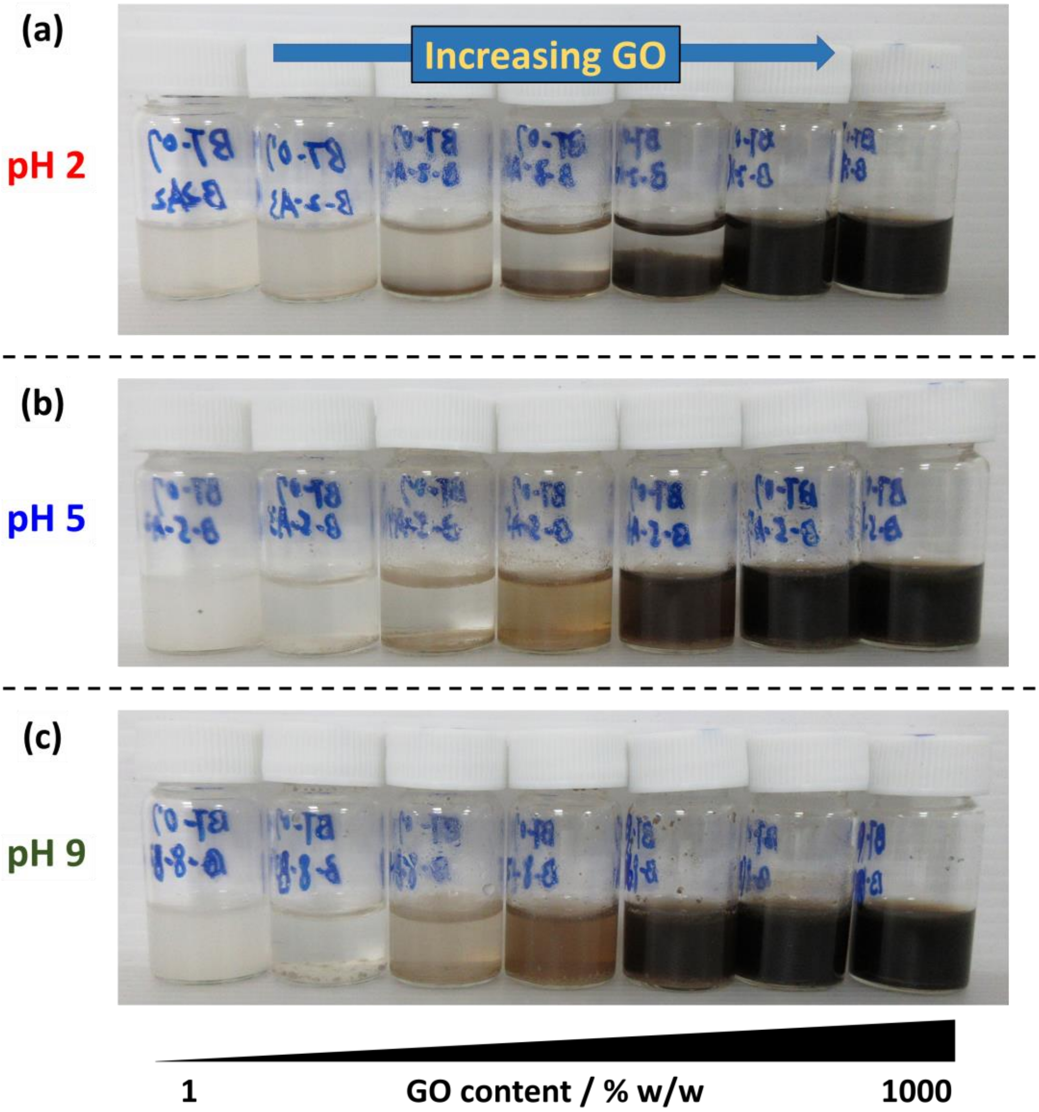

Figure S8. Digital photographs of vials containing PEGVP latex mixed with increasing quantities of GO nanosheets at (a) pH 2, (b) pH 5 and (c) pH 9 after standing overnight (entries 1–21, Table S2). The GO contents of the heteroflocculation dispersions from left to right are 1, 2, 10, 20, 100, 500 and 1000 % w/w relative to latex, respectively. The solids content was fixed at 0.1 % w/w.

Table S3. Summary of polymer/GO nanocomposite particles prepared *via* heteroflocculation between 139 nm V_{32}–B_{300} latex and GO nanosheets at varying pH in dilute aqueous solution at room temperature. The solids content was fixed at 0.1% w/w.

Entry	Solution pH	$D_{h,\ GO}$[a] / nm	GO content[b] / % w/w	Free GO[c] / %	Zeta potential[d] / mV
1	2	292	1		+34 ± 3
2	2	292	2		+33 ± 1
3	2	292	10	14	+26 ± 1
4	2	292	20	34	+23 ± 1
5	2	292	100		-6 ± 1
6	2	292	500	74	-19 ± 1
7	2	292	1000	75	-20 ± 0
8	5	111	1		+12 ± 3
9	5	111	2		-3 ± 2
10	5	111	10	9	-18 ± 5
11	5	111	20	5	-23 ± 1
12	5	111	100	74	-31 ± 1
13	5	111	500	98	-34 ± 1
14	5	111	1000	>99	-37 ± 1
15	9	103	1		-19 ± 9
16	9	103	2		-20 ± 2
17	9	103	10	4	-21 ± 3
18	9	103	20	50	-26 ± 2
19	9	103	100	>99	-34 ± 0
20	9	103	500	>99	-35 ± 2
21	9	103	1000	>99	-39 ± 2

[a] Mean hydrodynamic diameter of GO determined *via* DLS analysis.

[b] Concentration relative to latex.

[c] Mass fraction of free GO relative to GO added, as determined by UV-Vis analysis.

[d] Determined *via* aqueous electrophoresis analysis at the corresponding solution pH.

Table S4. Summary of polymer/GO nanocomposite particles prepared *via* heteroflocculation between 149 nm V_{67}–B_{300} latex and GO nanosheets at varying pH in dilute aqueous solution at room temperature. The solid content was fixed at 0.1% w/w.

Entry	Solution pH	$D_{h,\ GO}$[a] / nm	GO content[b] / % w/w	Free GO[c] / %	Zeta potential[d] / mV
1	2	292	1		+31 ± 1
2	2	292	2		+23 ± 1
3	2	292	10		+25 ± 1
4	2	292	20	60	+27 ± 1
5	2	292	100	5	+13 ± 3
6	2	292	500	62	+15 ± 1
7	2	292	1000	45	+17 ± 1
8	5	111	1		-14 ± 3
9	5	111	2		-17 ± 2
10	5	111	10	9	-25 ± 0
11	5	111	20	7	-29 ± 0
12	5	111	100	44	-31 ± 1
13	5	111	500	91	-37 ± 0
14	5	111	1000	>99	-35 ± 0
15	9	103	1	33	+12 ± 3
16	9	103	2	30	+8 ± 1
17	9	103	10	69	-14 ± 3
18	9	103	20	>99	-17 ± 5
19	9	103	100	>99	-33 ± 1
20	9	103	500	>99	-31 ± 1
21	9	103	1000	>99	-38 ± 1

[a] Mean hydrodynamic diameter of GO determined *via* DLS analysis.

[b] Concentration relative to latex.

[c] Mass fraction of free GO relative to GO added, as determined by UV-Vis analysis.

[d] Determined *via* aqueous electrophoresis analysis at the corresponding solution pH.

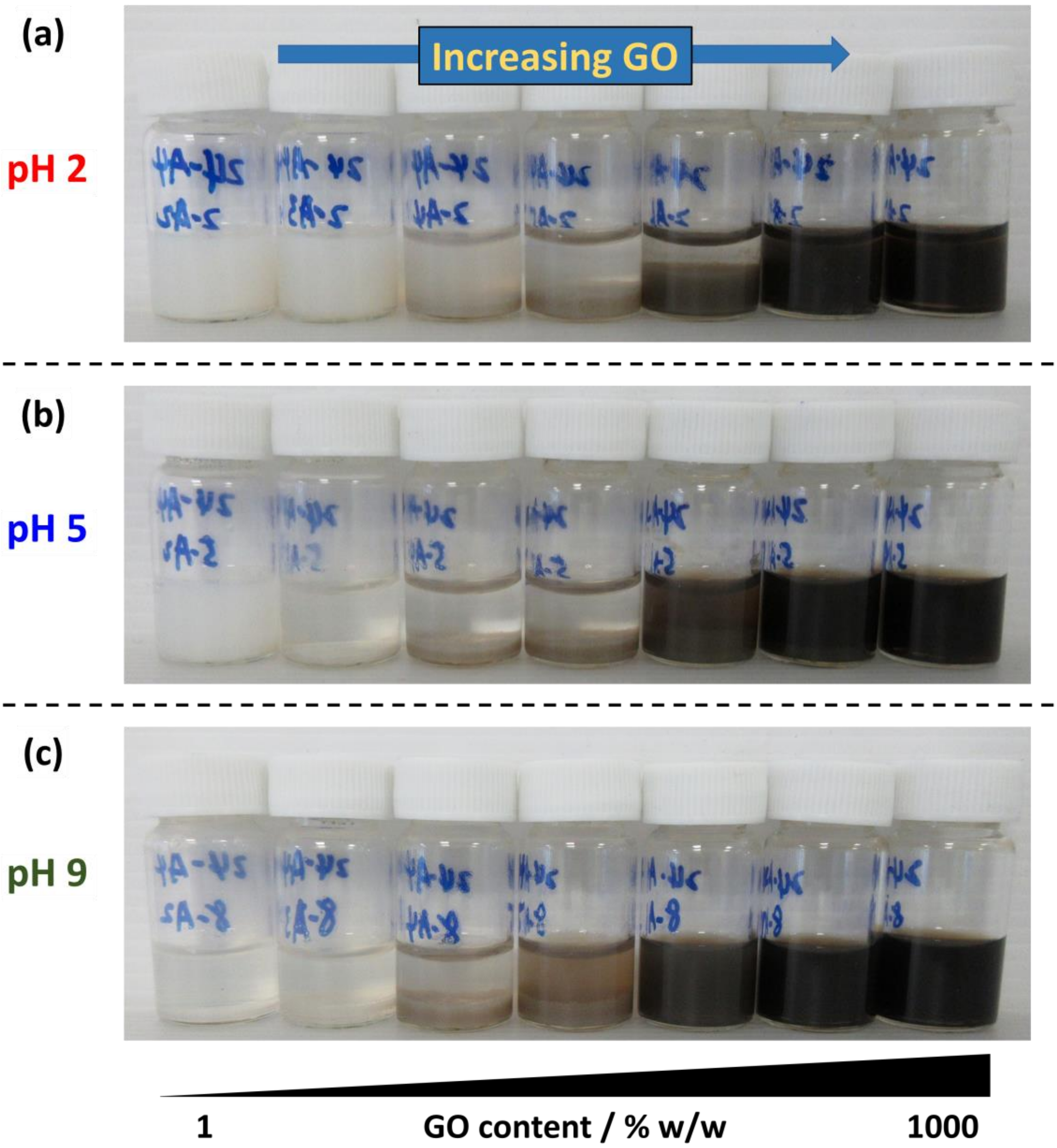

Figure S9. Digital photographs of vials containing V_{32}–B_{300} latex and increasing quantities of GO nanosheets at (a) pH 2, (b) pH 5 and (c) pH 9 after standing overnight (entries 1–21, Table S3). The GO contents of the heteroflocculation dispersions from left to right are 1, 2, 10, 20, 100, 500 and 1000 % w/w relative to latex, respectively. The solids content was fixed at 0.1 % w/w.

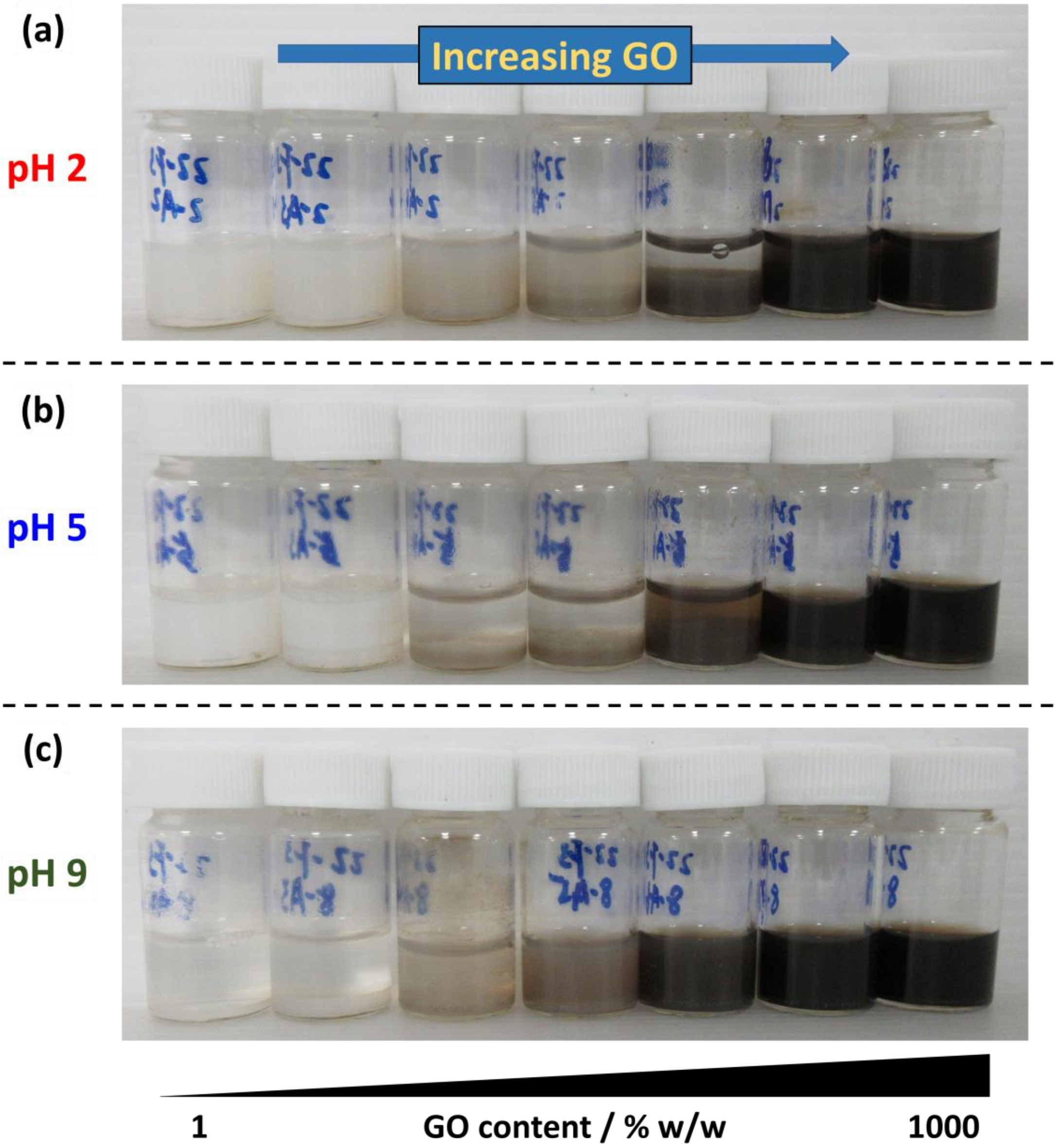

Figure S10. Digital photographs of vials containing V_{67}–B_{300} latex and increasing quantities of GO nanosheets at (a) pH 2, (b) pH 5 and (c) pH 9 after standing overnight (entries 1–21, Table S4). The GO contents of the heteroflocculation dispersions from left to right are 1, 2, 10, 20, 100, 500 and 1000 % w/w relative to latex, respectively. The solids content was fixed at 0.1 % w/w.

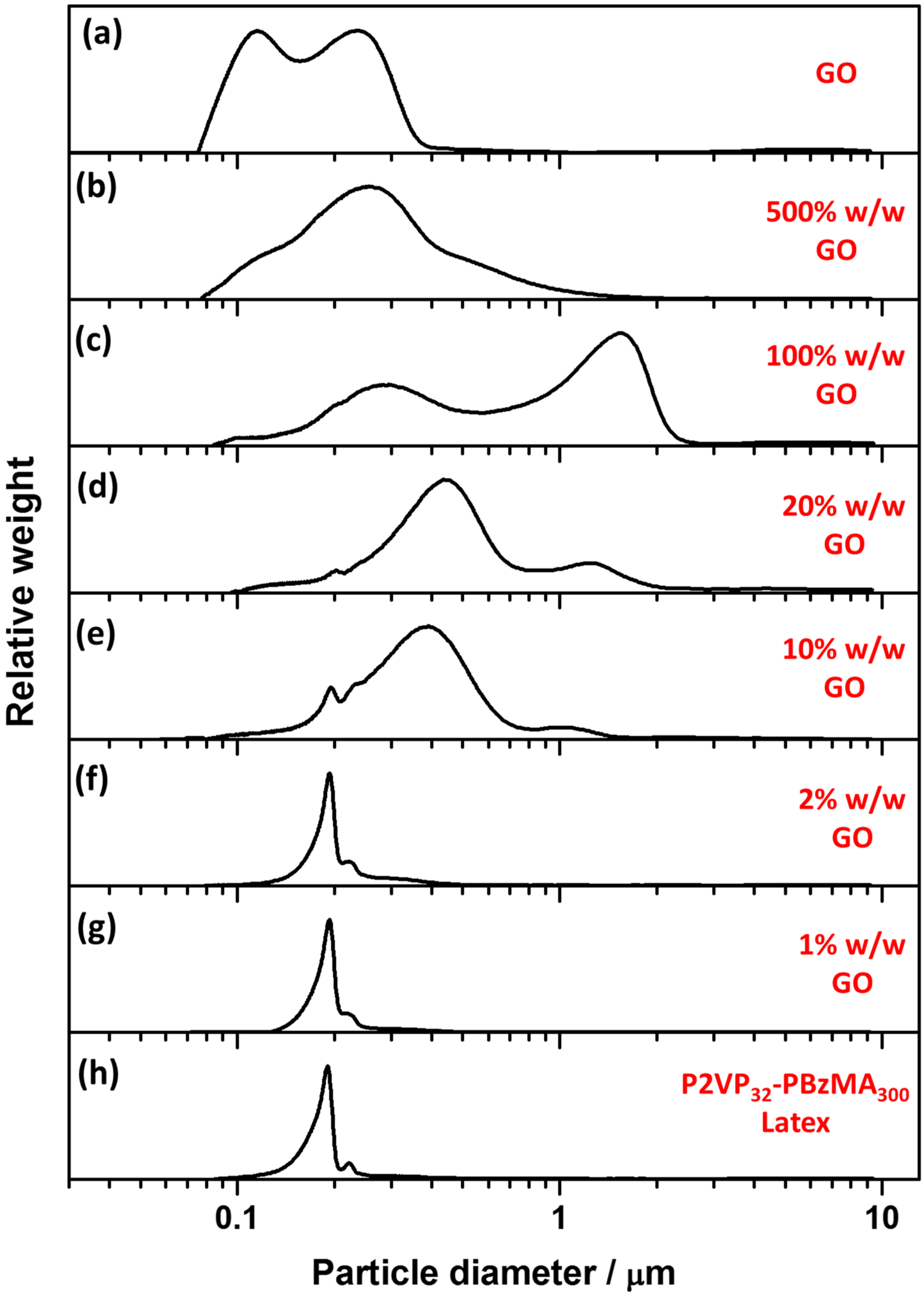

Figure S11. DCP particle size distributions obtained for V_{32}–B_{300}/GO nanocomposite particles prepared *via* heteroflocculation with varying GO content at pH 2. The density of the latex and nanocomposite particles was taken as 1.18 g cm^{-3}. (a) represents GO nanosheets obtained *via* sonication at 70% amplitude for 30 min. (b) – (g) represent entries 1–6 from Table S3, whereas (h) shows data obtained for entry 1 in Table 1.

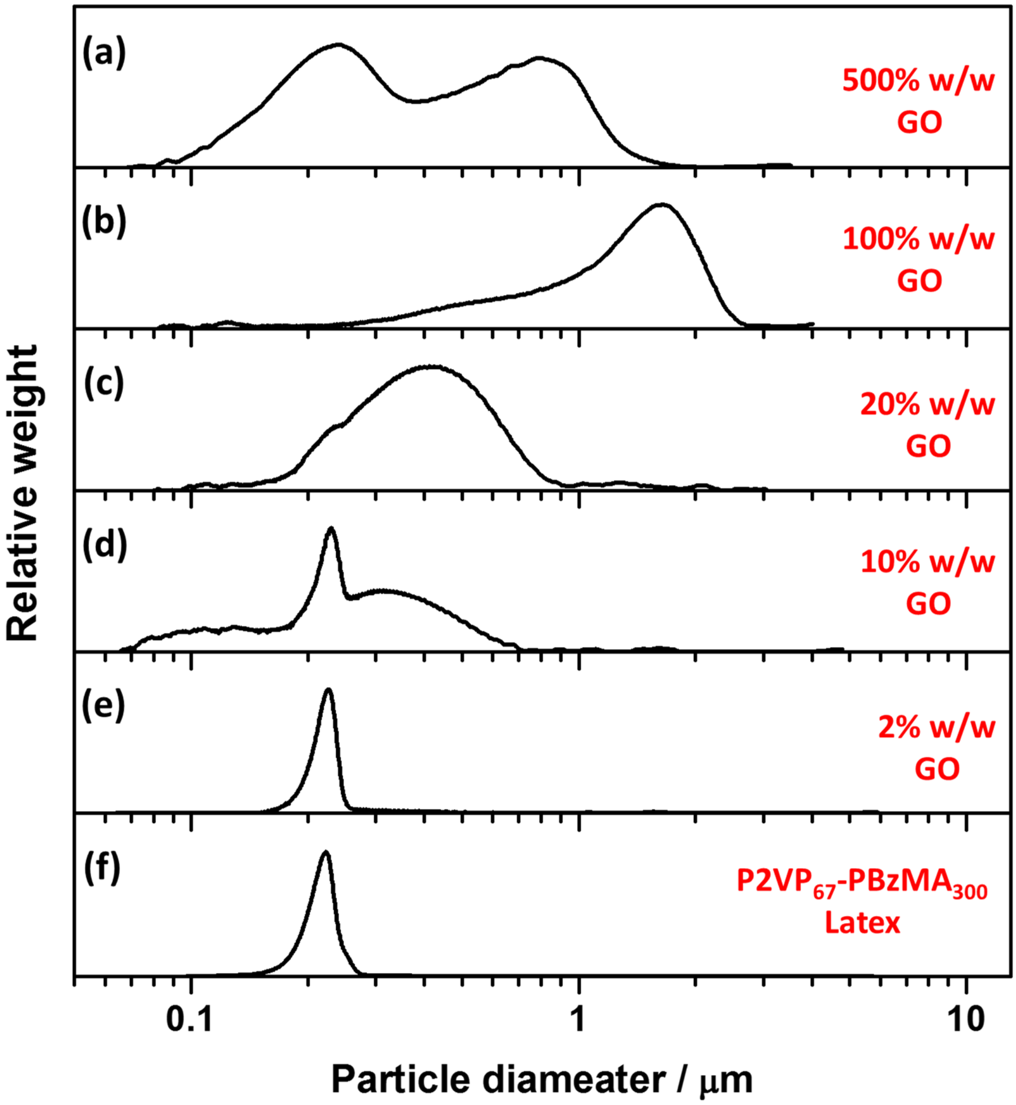

Figure S12. DCP particle size distributions obtained for V_{67}–B_{300}/GO nanocomposite particles prepared *via* heteroflocculation with varying GO content at pH 2. The density of the latex and nanocomposite particles was taken as 1.18 g cm^{-3}. (a) – (e) represent entries 2–6 from Table S4, whereas (f) shows data obtained for entry 2 in Table 1.

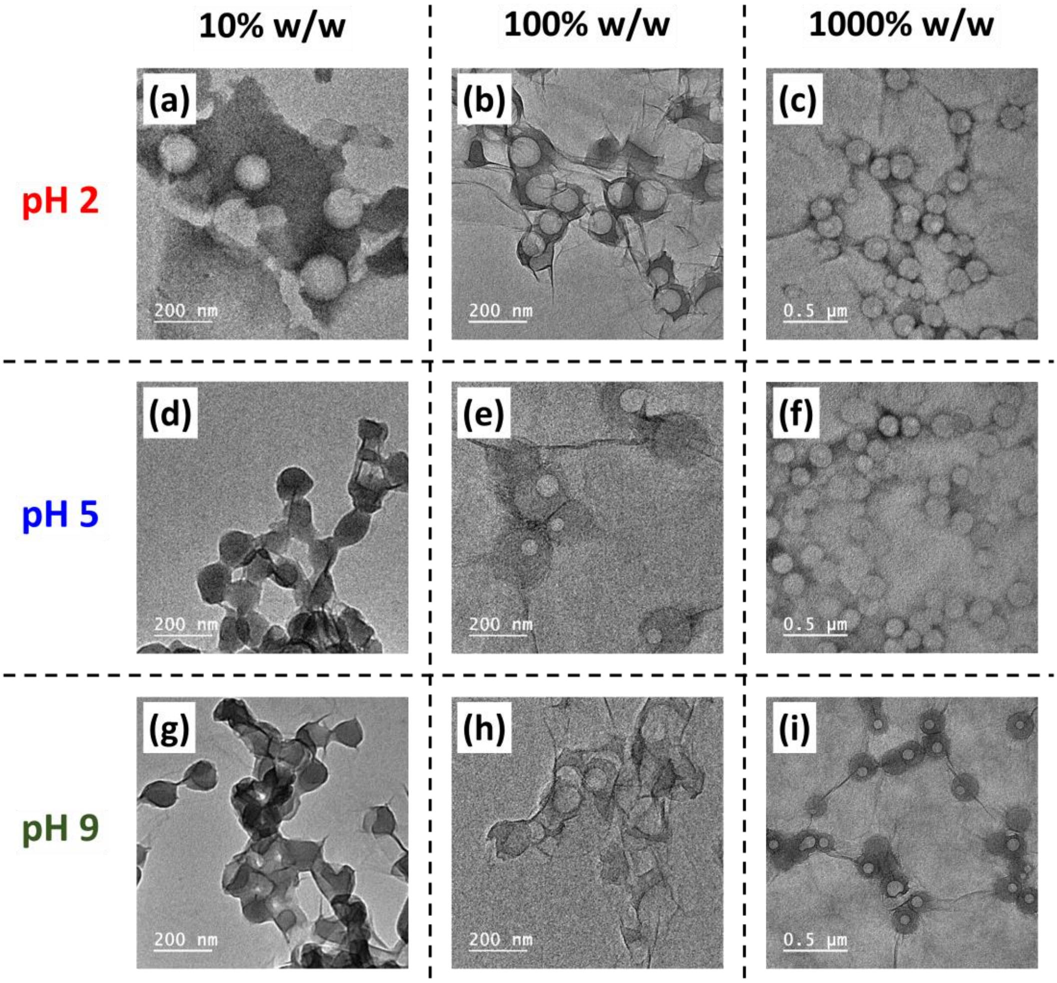

Figure S13. Representative TEM images for polymer/GO nanocomposite particles prepared *via* heteroflocculation between V_{67}–B_{300} latex and GO with varying content (10 % w/w, 100 % w/w and 1000 % w/w). Images (a–c), (d–f) and (g–i) correspond to heteroflocculation conducted in aqueous solution at pH 2, 5 and 9, respectively (entries 3, 5, 7, 10, 12, 14, 17, 19 and 21, Table S4).

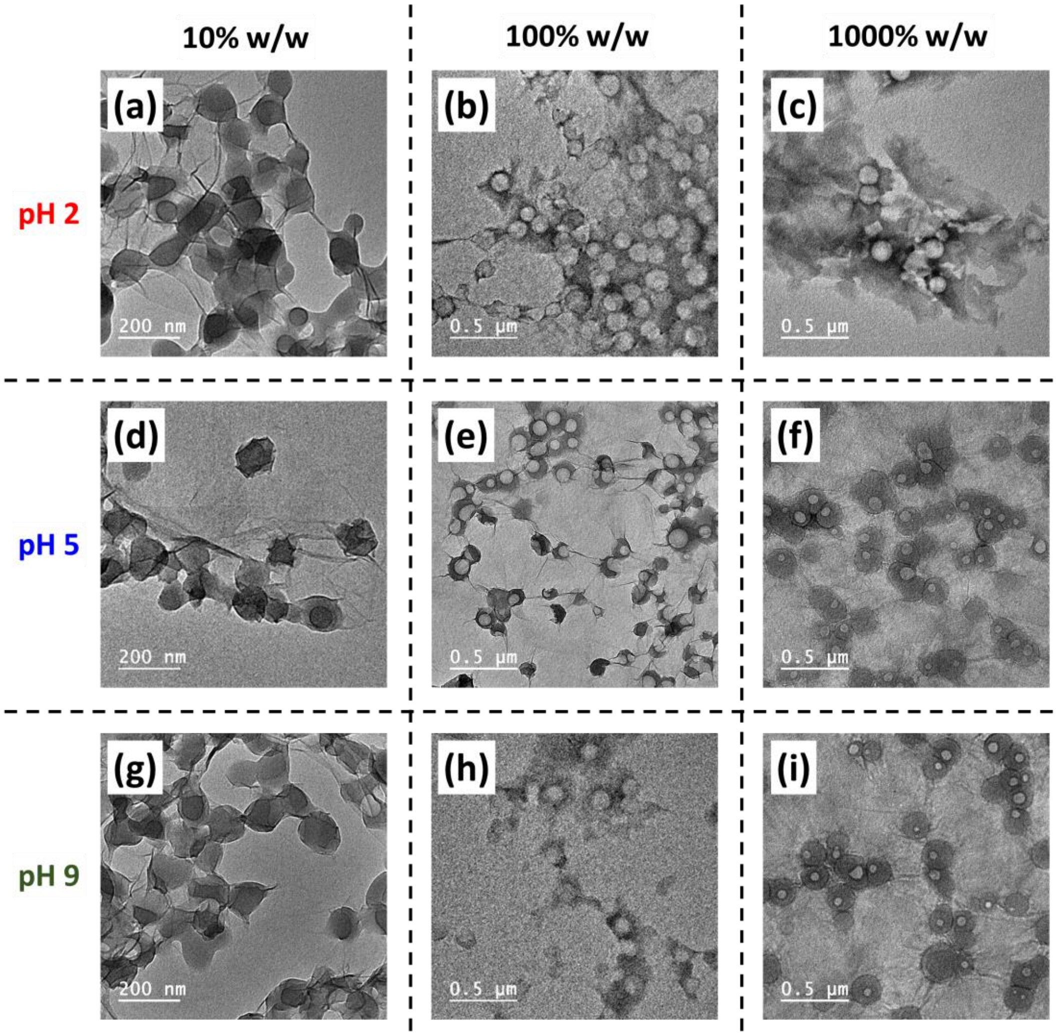

Figure S14. Representative TEM images for polymer/GO nanocomposite particles prepared *via* heteroflocculation between V_{32}–B_{300} latex and GO with varying content (10 % w/w, 100 % w/w and 1000 % w/w). Images (a–c), (d–f) and (g–i) correspond to heteroflocculation conducted in aqueous solution at pH 2, 5 and 9, respectively (entries 3, 5, 7, 10, 12, 14, 17, 19 and 21, Table S3).

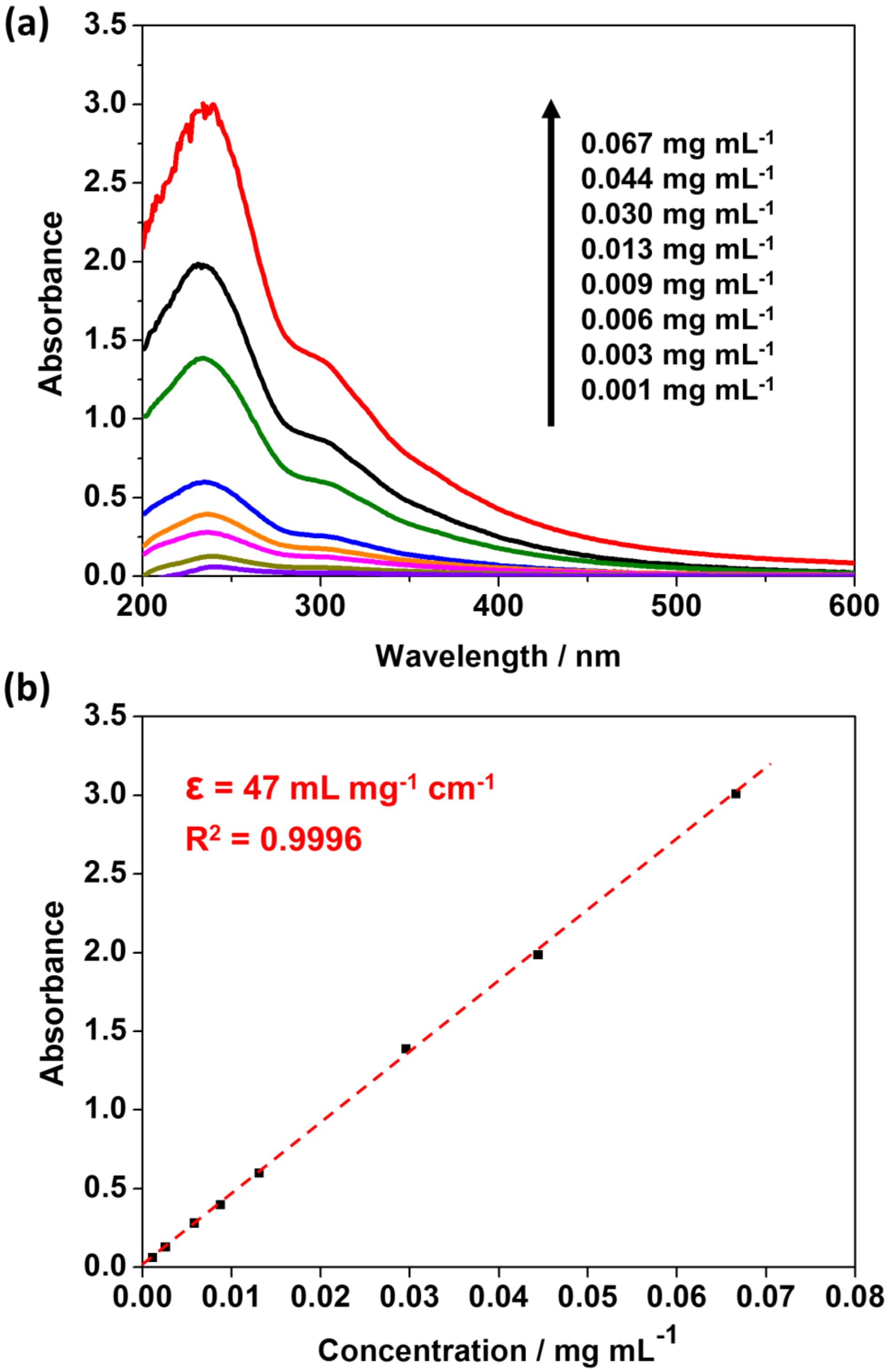

Figure S15. (a) UV-Vis spectra obtained for GO aqueous dispersions ranging from 0.001 to 0.067 mg mL^{-1} and (b) Beer-Lambert plot showing a linear relationship between the absorbance per unit path length and the GO concentration.

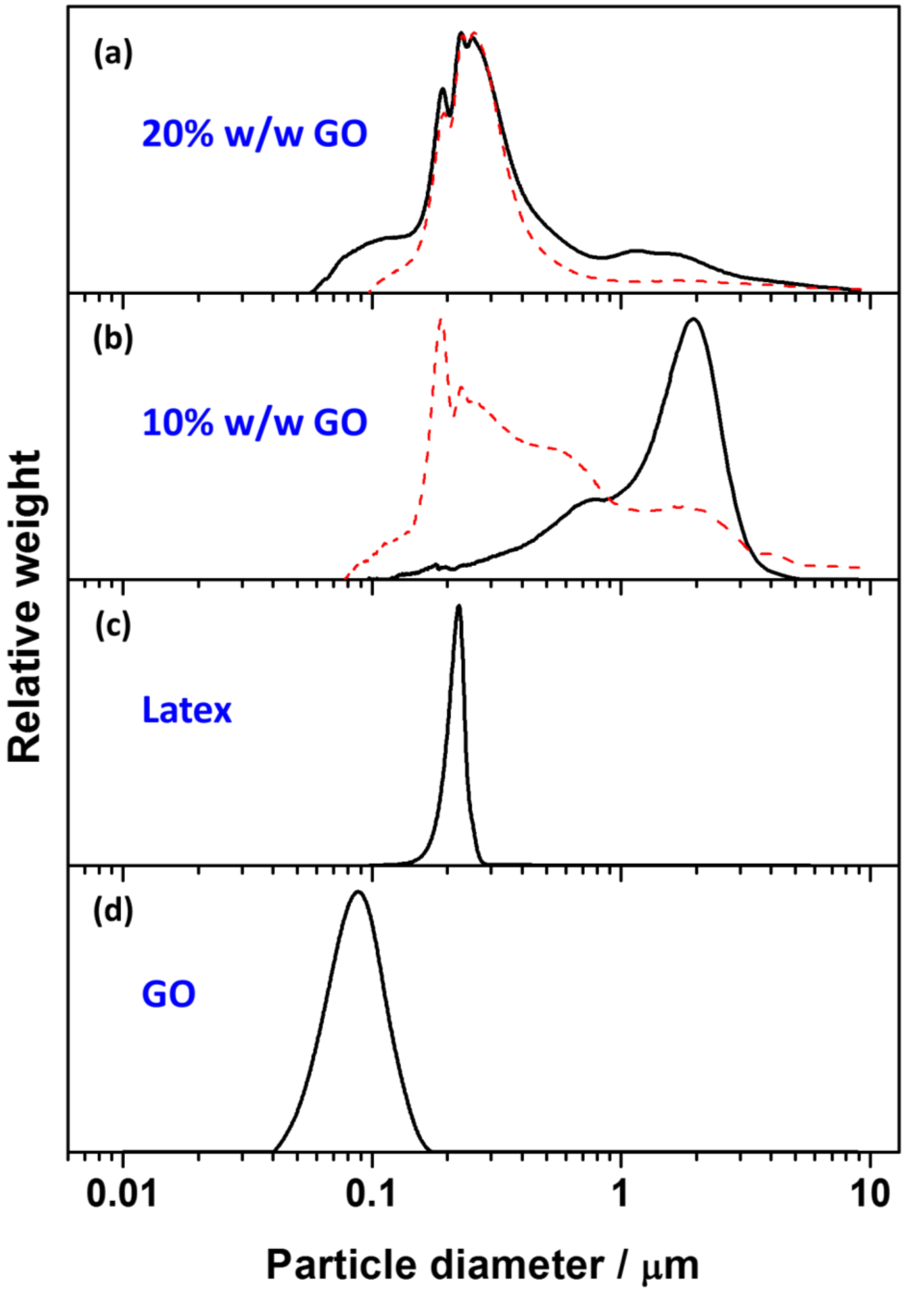

Figure S16. DCP particle size distributions obtained before (solid line) and after (dashed line) sonication for V_{67}–B_{300}/GO nanocomposite particles prepared at pH 5 with GO content of (a) 20 % w/w (entry 11, Table S4) and (b) 10 % w/w (entry 10, Table S4), (c) V_{67}–B_{300} latex (entry 2, Table 1) and (d) GO nanosheets obtained *via* sonication at 70 % amplitude for 30 min in aqueous solution at pH 5. For ease of comparison, the density used for all DCP analyses was fixed as 1.18 g cm^{-3}.

References

1. A. Lerf, H. He, M. Forster and J. Klinowski, *The Journal of Physical Chemistry B*, 1998, **102**, 4477-4482.
2. T. Nakajima and Y. Matsuo, *Carbon*, 1994, **32**, 469-475.
3. S.-P. Wen, Q. Yue and L. A. Fielding, *Polymer Chemistry*, 2021, **12**, 2122-2131.
